# HMGA2 regulates circular RNA ASPH to promote tumor growth in lung adenocarcinoma

**DOI:** 10.1038/s41419-020-2726-3

**Published:** 2020-07-27

**Authors:** Li Xu, Ye Ma, Hua Zhang, Qi-Jue Lu, Lie Yang, Ge-Ning Jiang, Wei-Lin Liao

**Affiliations:** 1 Department of Thoracic Surgery, Shanghai Pulmonary Hospital, Tongji University School of Medicine, Shanghai, China; 2 Department of Cardiothoracic Surgery, Changhai Hospital, Second Military Medical University, Shanghai, China; 3 Department of Thoracic Surgery, General Hospital of Western Theater Command, Chengdu, China; 4 Section of Science and Training, General Hospital of Western Theater Command, Chengdu, China

**Keywords:** Non-coding RNAs, Non-small-cell lung cancer

## Abstract

In this study, we identified a circular form of ASPH RNA (circASPH), expression of which was upregulated in lung adenocarcinoma and the human lung adenocarcinoma cell lines. We also found a positive correlation between circASPH level and the T and N stages of lung adenocarcinoma patients. Patients with higher levels of circASPH had a shorter overall survival. Moreover, we demonstrated that circASPH was directly regulated by HMGA2 and Twist1. The direct positive regulation of circASPH by Twist1 was dependent on the presence of HMGA2. Functional assays indicated that circASPH promoted the proliferation, migration, and invasion of lung adenocarcinoma cell lines in vitro. The promoting effect of tumor growth by circASPH was also observed in vivo. Mechanistically, circASPH was identified to act as a molecular sponge for miR-370 and abrogate miR-370-mediated inhibition of HMGA2. Finally, we demonstrated that the oncogenic function of circASPH was HMGA2-dependent. These findings reveal the oncogenic functions of the HMGA2-circASPH-HMGA2 axis and may be useful in developing circRNA-based therapeutic strategies for lung adenocarcinoma.

## Introduction

The high-mobility group AT-hook 2 (HMGA2) gene, a member of the chromatin remodeler family, has been identified as an oncogene in many malignancies^1–3^. During transcription, HMGA2 alters the structure of DNA and interacts with transcription factors to enhance the expression of downstream targets. The important roles of HMGA2 and its downstream genes in tumor progression have been demonstrated in previous studies^4,5^. One of our previous studies showed that HMGA2 enhanced cell proliferation and invasion in squamous cell lung cancer^6^. To date, the regulatory effects of HMGA2 on protein-coding oncogenes have been demonstrated by multiple studies. However, little is known about the effect of HMGA2 on noncoding RNAs, including circular RNAs (circRNAs).

CircRNAs are a naturally occurring family of noncoding RNAs that are highly represented in the eukaryotic transcriptome^7^. CircRNAs, characterized by covalently closed loop structures, are highly stable and resistant to degradation by RNases and other exonucleases compared with their linear counterparts, and are predominantly in the cytoplasm^8,9^. The functions of circRNAs in biological processes are being gradually unveiled, among which the well-known “sponge of miRNA” function was demonstrated by two high-quality studies^9,10^. Unlike other types of noncoding RNAs, several endogenous circRNAs can also be translated into proteins^11,12^.

It is becoming increasingly evident that dysregulated circRNAs are involved in almost all types of cancers, such as hepatocellular carcinoma, bladder cancer, breast cancer, and lung cancer, the latter having the highest morbidity and mortality rates in the world^13–18^. As the most common pathological classification of lung cancer, lung adenocarcinoma, which is often asymptomatic in the early stages, causes ~500,000 deaths each year^18^. Despite recent improvements in comprehensive therapies, the prognosis of lung adenocarcinoma remains unsatisfactory due to tumor recurrence and metastasis^18^. Elucidating the progression of lung adenocarcinoma is of critical importance for developing new therapeutic strategies.

In this study, we identified a circular form of aspartate beta-hydroxylase (ASPH) RNA (circASPH), which was upregulated in lung adenocarcinoma. We further demonstrated that circASPH, which was directly regulated by HMGA2 and Twist1, promoted malignant phenotypes in lung adenocarcinoma by sponging miR-370, and the oncogenic effects of circASPH were HMGA2-dependent.

## Materials and methods

### Patients and tissue samples

All primary lung adenocarcinoma tissues and adjacent normal tissues were collected from patients who had undergone surgery at the Department of Thoracic Surgery, General Hospital of Western Theater Command (Chengdu, China). All cancerous and matching adjacent normal tissue samples were initially histologically diagnosed at the Department of Pathology, General Hospital of Western Theater Command. All the study protocols were approved by the Institutional Review Boards of General Hospital of Western Theater Command. For examinations utilizing human lung tissue specimens, all patients gave written informed consent.

### RNA-sequencing (RNA-seq) analysis

CircRNA high-throughput sequencing and subsequent bioinformatics analysis were performed, as described in Supplementary File 1. The raw circRNA-seq data are deposited in the SRA database [PRJNA555772 (SRP215712)].

### Immunohistochemical (IHC) staining

The paraffin-embedded tissues from BALB/c nude mice were cut into 4-μm slices. The immunohistochemical staining was performed as described elsewhere^19^. The primary antibody used was anti-HMGA2 (1:500, PA5-21320; Invitrogen).

### Migration and invasion assays

The migration assay was performed as described elsewhere^6^. Images were taken at 0 and 36 h. The migration distance was calculated as the difference in the gap width between the two imaging time points. The invasion assay was performed as described elsewhere^20^. After incubation at 37 °C for 24 h, the cells in the upper chamber were removed by a cotton swab, and the cells that had traversed the membrane were fixed, stained in a 0.1% crystal violet solution, and counted.

### Scanning electron microscopy assay

The scanning electron microscopy assay was performed according to a previous study^21^ with modifications (Supplementary File 1).

### RNA fluorescence in situ hybridization (RNA FISH)

RNA FISH was performed as described previously^22^. The sequences of probes used in the FISH assay were as follows: Cy3-labeled circASPH probe 5′-TCTCTCTTTAAGTCCTTTTGCTTTTTGTTC-3′; and Dig-labeled locked nucleic miR-370 probe 5′-ACCAGGTTCCACCCCAGCAGGC-3′.

### Statistical analysis

Data are presented as the mean ± SD. Statistical comparisons were analyzed with SAS software, version 9.4 (SAS Institute). The quantitative data were evaluated for normal distribution by the Kolmogorov–Smirnov *Z* test. Statistically significant differences were calculated using Student’s *t* test, nonparametric Mann–Whitney *U* test, Pearson’s correlation, and Kaplan–Meier analysis, as appropriate. All *P*-values were two-sided, and a value <0.05 was considered to indicate a significant difference.

Other methods used in this study are described in Supplementary File 1.

## Results

### Profiling of HMGA2-related circRNAs and the expression levels of candidate circRNAs

**Fig. 1:**
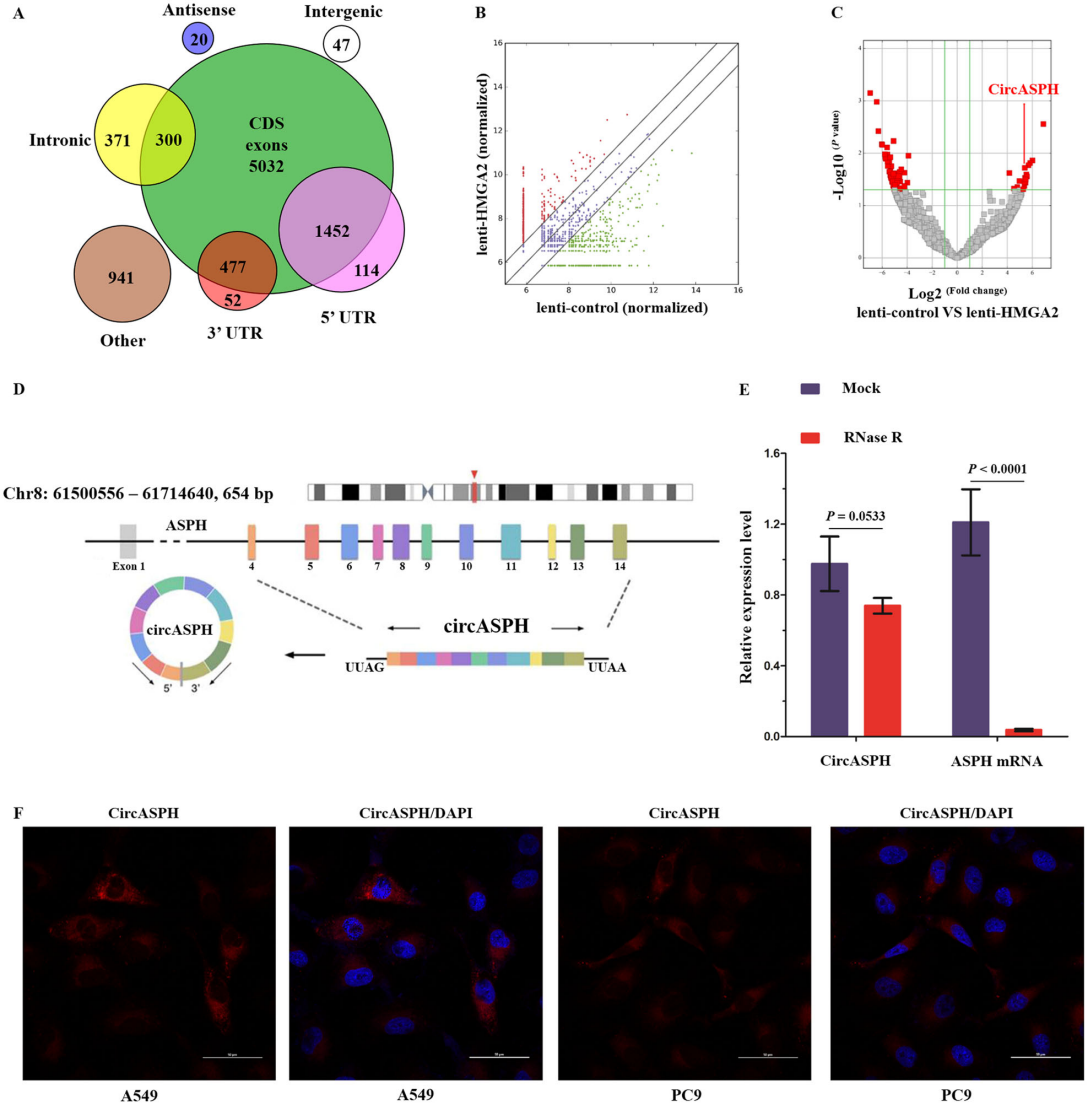
Profiling of HMGA2-related circRNAs and characteristics of circASPH. **a** Venn plot shows the number of circRNAs derived from different genomic regions. **b**, **c** Scatter plot and volcano plot visualize the difference between circRNAs expressions in the lenti-control and lenti-HMGA2 A549 cells, respectively. **d** Illustration of the annotated genomic region of circASPH. **e** qRT-PCR for the abundance of circASPH and ASPH mRNA in the total RNA from A549 cells with RNase R treatment. **f** FISH indicates the localization of circASPH in A549 and PC9 cells. *n* = 3 for each cell group.

An increasing number of studies have demonstrated the oncogenic roles of HMGA2 and its downstream target genes in malignancies^4,5^. However, little is known about the regulation of circRNAs by HMGA2 in malignancies. To investigate the downstream circRNAs of HMGA2 in lung adenocarcinoma, we first used a lentivirus vector to stably overexpress HMGA2 in lung adenocarcinoma cell line A549 (Supplementary Fig. S1a–c). Next, we characterized circRNA transcripts using RNA-seq analysis of linear and ribosomal RNA-depleted total RNA from lenti-HMGA2- and lenti-control-infected A549 cells. As a result, a total of 6576 circRNAs were identified, and 4477 of them were annotated in Circbase. Analysis of the 6576 circRNAs and their host genes indicated that one gene could produce multiple circRNAs (6576 circRNAs from 3362 genes). Most of the 6576 circRNAs were derived from exons (Fig. 1a). The expressions of 18 circRNAs were significantly upregulated by HMGA2 overexpression, while the expressions of 69 circRNAs were significantly downregulated by HMGA2 overexpression (Fig. 1b, c).

Among the ten most upregulated circRNAs from the sequencing results, we chose five circRNAs (circCDR1, circTRIO, circASPH, circNETO2, and circPTGR1) with the lowest *P*-values to verify in the lenti-HMGA2 A549 cells. CircCDR1, circNETO2, and circASPH were the three most upregulated circRNAs among the five candidate circRNAs (Supplementary Fig. S1d). We further investigated the levels of circCDR1, circNETO2, and circASPH in 42 pairs of lung adenocarcinoma and adjacent normal tissues (Supplementary Table 1 and Supplementary Fig. S1f–h). Among the three circRNAs, one of the special importance was the circASPH (Supplementary Fig. S1g). We also found that the levels of circASPH were significantly higher in A549 cells and PC9 cells than in the normal human bronchial epithelial cell line BEAS-2B (Supplementary Fig. S1e). CircASPH (circbase ID: hsa_circ_0084606), derived from exon 4 to exon 14 of the ASPH gene (Fig. 1d), was resistant to digestion by RNase R (Fig. 1e). To demonstrate the localization of circASPH, we designed a probe against the circASPH junction and conducted a RNA FISH assay. As shown in Fig. 1f and Supplementary Fig. S2a, circASPH was stable and abundant in the cytoplasm of A549 and PC9 cells. Cancerous tissues had a higher level of circASPH.

### Correlation between circASPH expression level and clinical characteristics of lung adenocarcinoma

The association between circASPH and clinical and pathologic parameters was further evaluated (Supplementary Table 1). Patients with larger tumor sizes exhibited higher expression levels of circASPH (Supplementary Fig. S2b). Patients with tumor-node metastasis stage II–III exhibited higher circASPH levels than patients with stage I metastasis (Supplementary Fig. S2c). Kaplan–Meier survival curves showed that patients with higher levels of circASPH had a shorter overall survival (Supplementary Fig. S2d).

### HMGA2 cooperates with Twist1 to directly regulate circASPH expression

To verify the upregulation of circASPH by HMGA2, we also used qRT-PCR to detect the expression levels of circASPH in stable HMGA2-overexpressing PC9 cells (Supplementary Fig. S3a–c). The results showed that overexpression of HMGA2 significantly enhanced circASPH expression (Supplementary Fig. S3d). Taking into account the chromatin remodeling effect of HMGA2 during transcription, we postulated that the ASPH gene might be a direct downstream gene of HMGA2. We further conducted a chromatin immunoprecipitation (ChIP) assay to examine whether HMGA2 was recruited to the ASPH promoter. An enrichment of HMGA2 was observed in the region from ~ −336 to −1147 base pairs (bp) upstream of the transcription start site (TSS) of ASPH (Supplementary Fig. S3e).

Chromatin remodeling factors commonly cooperate with transcription factors to regulate downstream genes. Several transcription factors play important roles in human cancer^23^. To determine the transcription factor cooperating with HMGA2, a co-IP assay in A549 cells using anti-HMGA2 antibody was performed, followed by a mass spectrometry assay. As shown in Supplementary Fig. S3f, STAT3, Twist1, and BTF3 could be transcription factors interacting with HMGA2. Emerging evidence suggests that Twist1, a basic helix–loop–helix transcription factor, is an important oncogene promoting the progression of lung cancer^24,25^. According to Meng et al., in addition to protein-coding mRNAs, circRNAs are also direct downstream targets of Twist1. Twist1 directly upregulates circ-10720^21^. Therefore, western blotting of the co-IP eluate was conducted to validate the endogenous interaction between the HMGA2 and Twist1 proteins in A549 cells (Supplementary Fig. S3g).

To detect how Twist1 regulates circASPH, we transfected A549 cells with Ad-Twist1 to overexpress Twist1 (Supplementary Fig. S4a–c). Notably, Twist1 overexpression upregulated the level of circASPH (Supplementary Fig. S4d). To identify whether Twist1 was recruited to the promoter of ASPH, a ChIP assay was performed using a Twist1-specific antibody in A549 cells. We found an enrichment of Twist1 in the region from −30 to −285 bp, upstream of ASPH TSS (Supplementary Fig. S4m), which was located near the binding site of HMGA2.

To determine whether the regulation of circASPH by Twist1 was HMGA2-dependent, we transfected A549 cells with a lentiviral vector carrying HMGA2 siRNA to stably knock down HMGA2 (Supplementary Fig. S4e–g). The levels of circASPH were downregulated in lenti-siHMGA2 A549 cells (Supplementary Fig. S4h). Next, Ad-Twist1 was used to overexpress Twist1 in HMGA2 knockdown A549 cells (Supplementary Fig. S4i–k). By using qRT-PCR, we found that the enhancement of circASPH expression was not notable in Ad-Twist1/lenti-siHMGA2 A549 cells compared with the Ad-control/lenti-siHMGA2 group (Supplementary Fig. S4l). A ChIP assay was further performed in lenti-siHMGA2 A549 cells. The results showed that the recruitment of Twist1 to the ASPH promoter was decreased by HMGA2 knockdown (Supplementary Fig. S4m). Taken together, these data suggested that circASPH was directly regulated by HMGA2 and Twist1, and the direct positive regulation of circASPH by Twist1 was dependent on the presence of HMGA2.

We also investigated the regulatory effects of HMGA2 and Twist1 on the host gene of circASPH. We found that the expression levels of ASPH pre-mRNA were significantly increased in lenti-HMGA2 and Ad-Twist1 A549 cells compared with control cells (Supplementary Fig. S4n, o). However, the levels of mature ASPH mRNA were not significantly affected by HMGA2 and Twist1 overexpression (Supplementary Fig. S4n, o).

### CircASPH promotes tumorigenesis of lung adenocarcinoma

The findings that circASPH was upregulated in lung adenocarcinoma tissues, and was directly regulated by HMGA2 and Twist1 prompted us to consider circASPH as an oncogene in lung adenocarcinoma. To verify this hypothesis, we introduced siRNA specifically targeting the junction site to knock down circASPH expression in A549 cells (Supplementary Fig. S5a, b) since circASPH levels are normally high in A549 cells. qRT-PCR revealed no obvious effect of circASPH knockdown on the level of ASPH mRNA (Supplementary Fig. S5b). Next, cell proliferation, migration, and invasion were examined by EdU, wound scratch, and transwell-invasion assays, respectively. Our data showed that the proliferation, migration, and invasion abilities were significantly decreased in A549 cells transfected with circASPH siRNA (Supplementary Fig. S5c–h). However, the morphology of A549 cells did not significantly change by circASPH knockdown (Supplementary Fig. S5i).

**Fig. 2:**
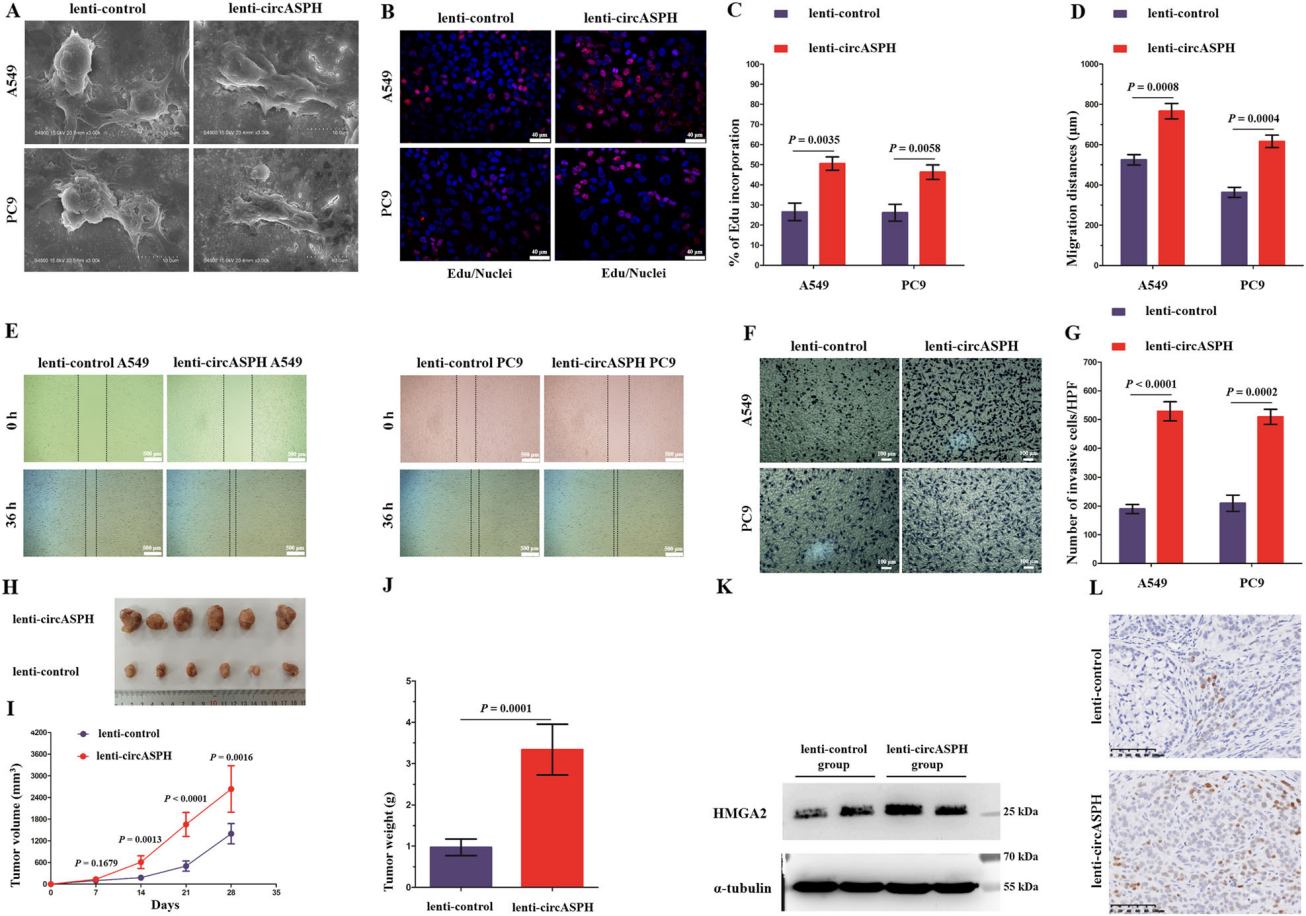
The oncogenic effects of circASPH in vitro and in vivo. **a** The morphologic changes in A549 and PC9 cells overexpressing circASPH were observed using a scanning electron microscopy assay. **b**, **c** DNA synthesis assessed using an EdU assay in the lenti-circASPH cells and control cells. Nuclei were stained with Hoechst 33342 (blue). The quantified data are presented as the percentage of EdU-incorporated cells. Log2 transformation was applied to deal with the ratio results and to obtain the normally distributed data. Then, *T* test was used to compare the difference between groups. **d**, **e** The migration of A549 and PC9 cells was significantly increased by circASPH overexpression. The migration distances (μm) of A549 and PC9 cells were quantified. **f**, **g** The invasion of A549 and PC9 cells was apparently increased by circASPH overexpression. The quantified data are presented as the number of invading cells per HPF. HPF high-power field. **h**–**j** Xenograft tumor models show that tumors grown from the circASPH-overexpressing cells were bigger than those grown from the control cells. **k** Western blot analysis of the levels of HMGA2 protein in the xenograft tumors. **l** IHC staining of the HMGA2 protein in the xenograft tumors. Every cell test was carried out in triplicate, and *n* = 3 for each cell group.

To further explore the oncogenic effects of circASPH, circASPH was overexpressed in A549 and PC9 cells via a lentiviral vector (Supplementary Fig. S5j, k). qRT-PCR showed that overexpression of circASPH did not apparently affect the ASPH mRNA level (Supplementary Fig. S5j, k). The morphological changes in A549 and PC9 cells overexpressing circASPH were documented using scanning electron microscopy. Compared with the control group, exogenous circASPH expression resulted in increased formation of cell pseudopodia and cells transforming into a mesenchymal phenotype (Fig. 2a). Next, the proliferative, migratory, and invasive capacities were also examined in vitro. As depicted in Fig. 2b to g, the upregulation of circASPH significantly promoted the proliferation, migration, and invasion of A549 and PC9 cells, suggesting a more aggressive phenotype. We further investigated the effect of circASPH on tumor growth in vivo. A total of 5 × 10^7^ circASPH-overexpressing A549 cells and negative control A549 cells were subcutaneously injected into the axilla of female athymic BALB/c nude mice. The results demonstrated that circASPH overexpression markedly increased tumor weight and volume compared with the negative controls (Fig. 2h–j). Western blot and IHC assays were performed for further inspection of the generated xenografts. Interestingly, we found that HMGA2 protein expression was remarkably elevated in the tumors formed by lenti-circASPH cells (Fig. 2k, l), indicating that circASPH might in turn enhance HMGA2 expression.

### CircASPH acts as a molecular sponge for miR-370

**Fig. 3:**
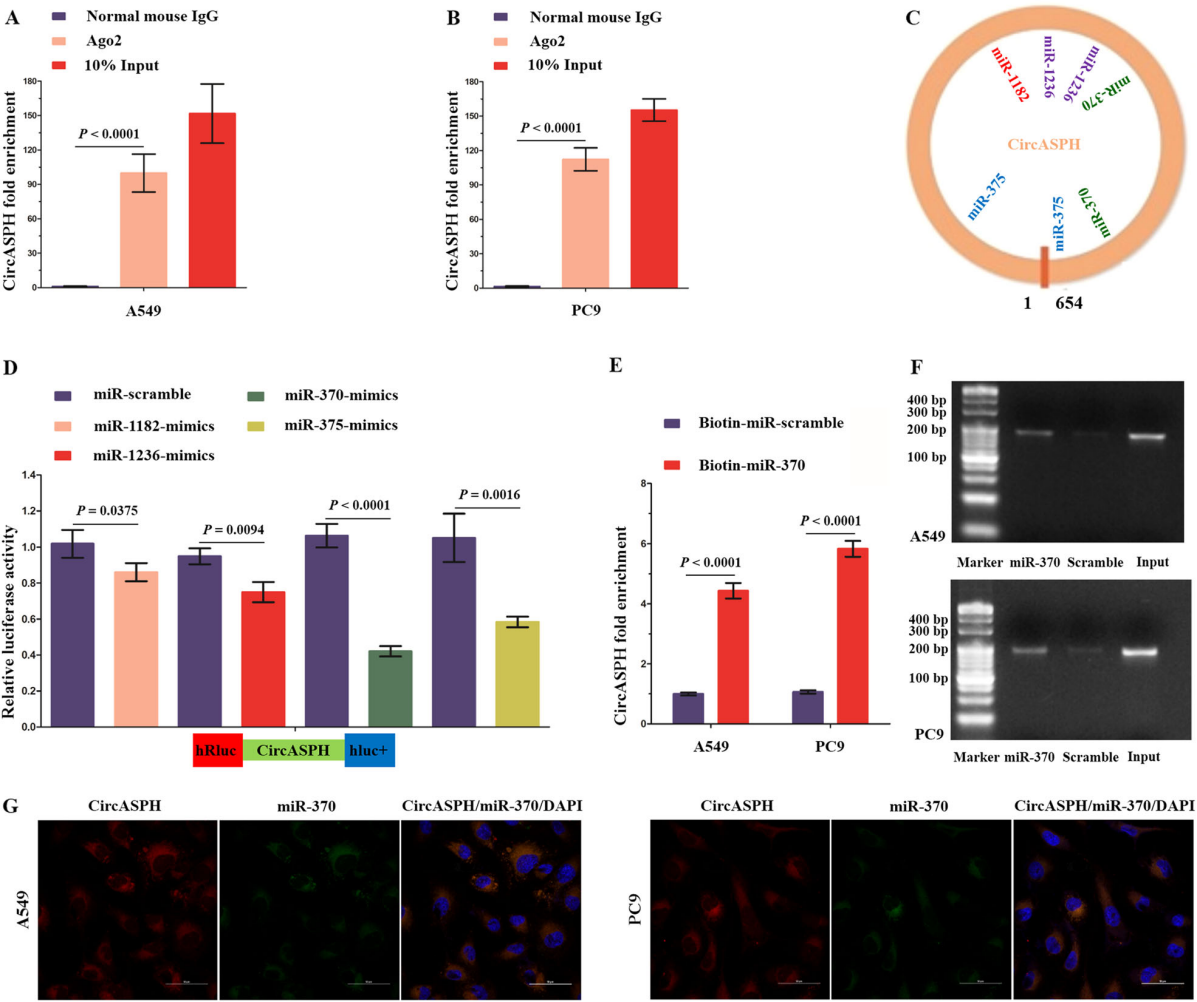
CircASPH acts as a sponge for miR-370. **a**, **b** Ago2 RIP assay for the amounts of circASPH in A549 and PC9 cells, respectively. The expression levels of circASPH were measured by RT-PCR. **c** Schematic model shows the potential binding sites of miR-1182, miR-1236, miR-370, and miR-375 on circASPH. **d** The whole mature sequence of circASPH was cloned into the downstream region of hRluc luciferase reporter gene. Dual-luciferase reporter assay analysis of the effects of miR-1182, miR-1236, miR-370, and miR-375 on circASPH in 293T cells. **e**, **f** Biotin-coupled miR-370 captured a fold change of circASPH in the complex as compared with biotin-coupled miR-Scramble. The captured complex from biotin-coupled miRNA capture assays were detected using qRT-PCR and semi-qPCR. **g** Co-localization between circASPH and miR-370 was observed in A549 and PC9 cells. Nuclei were stained with DAPI (blue). Every cell test was carried out in triplicate and *n* = 3 for each cell group.

Given that circRNAs have been shown to function as miRNA sponges^9,22^ and that circASPH is localized in the cytoplasm of A549 and PC9 cells, we speculated that circASPH promoted the progression of lung adenocarcinoma by sponging miRNAs. According to Starbase v2.0, circASPH has two potential binding sites for Ago2 protein on its mature sequence. To validate this prediction, we conducted a RNA-binding protein immunoprecipitation assay in A549 and PC9 cells to investigate the interaction between Ago2 protein and circASPH. By qRT-PCR, the specific enrichment of endogenous circASPH in Ago2 immunoprecipitates was detected (Fig. 3a, b), indicating that circASPH had miRNA-related functions. By a Starbase v2.0 prediction, we found that circASPH shared potential miRNA response elements for multiple miRNAs (Fig. 3c). We chose miR-1182, miR-1236, miR-370, and miR-375 for further study. To explore the effects of circASPH on these four candidate miRNAs, we constructed a psiCHECK-2 luciferase vector containing the whole region of circASPH and cotransfected this vector with miRNAs mimics into 293T cells (Fig. 3d). Compared with miR-370, the extents of miR-1182, miR-1236, and miR-375 impairing the luciferase activity of the reporter were relatively limited (Fig. 3d). FISH analysis showed that circASPH was colocalized with miR-370 in the cytoplasm of A549 and PC9 cells (Fig. 3g). To further confirm that circASPH directly binds to miR-370, biotin-coupled miRNA capture assays were performed. It was found that biotin-coupled miR-370 mimics captured significantly more circASPH than biotin-coupled miR-scramble (Fig. 3e, f). These data demonstrated that circASPH served as a molecular sponge of miR-370 in A549 and PC9 cells.

### MiR-370 is a tumor suppressor in lung adenocarcinoma

An earlier study showed that miR-370 acts as a tumor suppressor in bladder cancer^26^. According to Li et al., miR-370 mediates the tumor suppressing function of circITGA7 in colorectal cancer^27^. To investigate the role of miR-370 in lung adenocarcinoma, we first examined the expression levels of miR-370 in lung adenocarcinoma tissue samples (Supplementary Table 1). The results showed that cancerous tissues had a lower level of miR-370 (Supplementary Fig. S6a). We also found that the level of miR-370 was significantly lower in A549 and PC9 cells than in BEAS-2B cells (Supplementary Fig. S6b). We further explored the functions of miR-370 in A549 and PC9 cells by transfecting A549 and PC9 cells with miR-370-mimics (Supplementary Fig. S6c). EdU, wound scratch, and transwell-invasion assays were conducted using miR-scramble as a negative control, which indicated that miR-370 overexpression significantly inhibited A549 and PC9 cell proliferation, migration, and invasion (Supplementary Fig. S6d–j).

### CircASPH modulates the expression of HMGA2, a direct target of miR-370

**Fig. 4:**
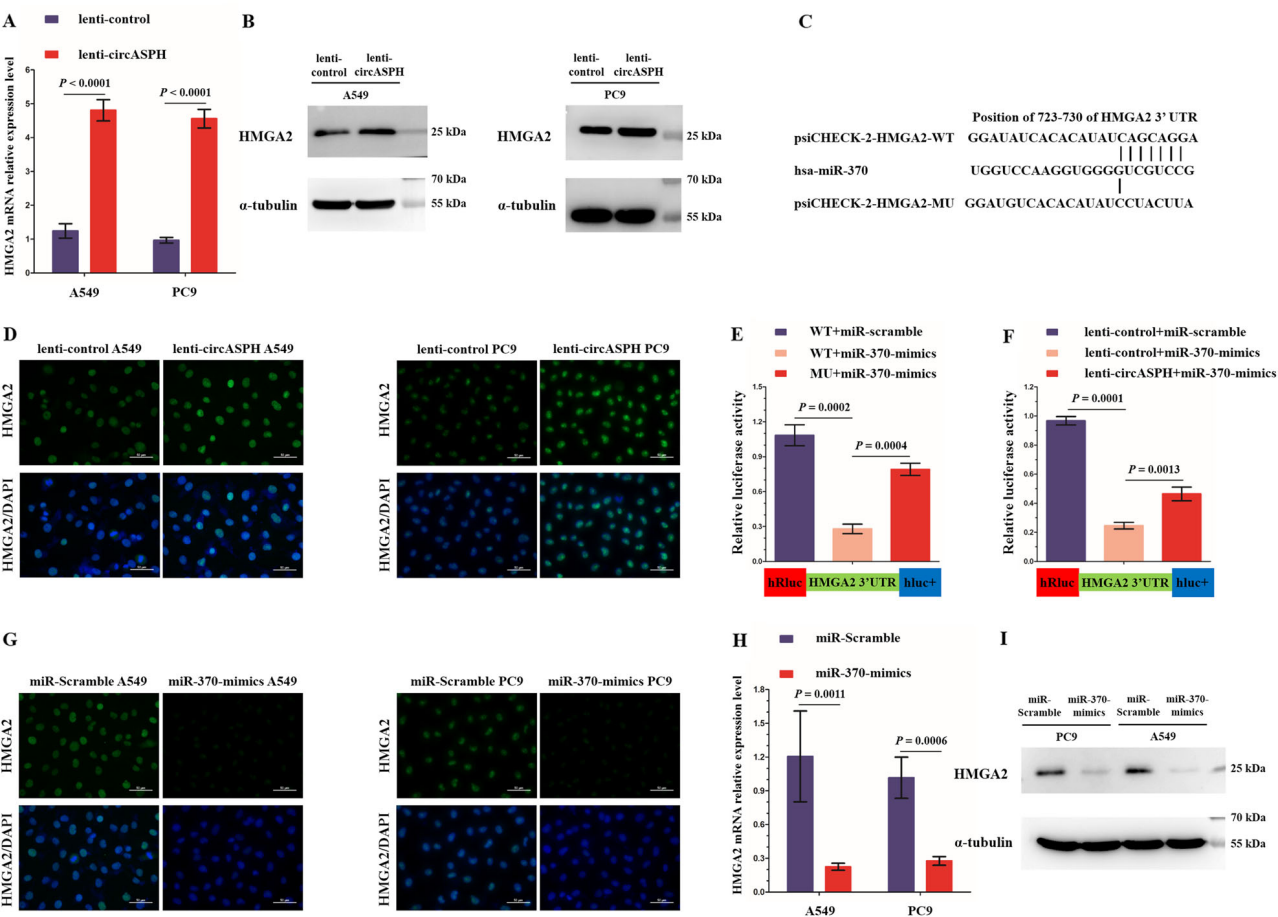
CircASPH abrogates miR-370-mediated inhibition of HMGA2. **a**, **b**, **d** The expression levels of both HMGA2 mRNA and protein were upregulated in the circASPH-overexpressing A549 and PC9 cells. **c** The predicted target site of miR-370 on HMGA2 mRNA 3′-UTR and the schematic diagram of site-directed mutations of the predicted target site. **e** Dual-luciferase reporter assay analysis of the effect of miR-370 on the 3′-UTR activity of HMGA2 in 293T cells. **f** Dual-luciferase reporter assay analysis of the effect of miR-370 on the 3′-UTR activity of HMGA2 in the circASPH-overexpressing and control 293T cells. **g**–**i** The expression levels of both HMGA2 mRNA and protein were significantly decreased in the miR-370-overexpressing A549 and PC9 cells. Every cell test was carried out in triplicate, and *n* = 3 for each cell group.

Considering the present findings that HMGA2 protein was significantly elevated in lenti-circASPH xenografts, we overexpressed circASPH in A549 and PC9 cells and examined HMGA2 levels in vitro. The results revealed that HMGA2 expression was significantly upregulated and aggregated in the nucleus of lenti-circASPH cells compared with the negative controls (Fig. 4a, b, d). Since circASPH was determined to be a sponge of miR-370, we postulated that circASPH positively regulated HMGA2 by sponging miR-370. Generally, miRNAs regulate the posttranscriptional expression of target genes by specifically binding to the 3′-untranslated regions (3′-UTRs)^28^. Using TargetScan, we found a potential target site of miR-370 on the 3′-UTR of HMGA2 mRNA (Fig. 4c). Next, we cloned the wild-type and mutant (predicted miR-370 binding site was mutated) 3′-UTR of HMGA2 mRNA into the psiCHECK-2 vector and performed a dual-luciferase reporter assay (Fig. 4e). We found that mutations of the predicted site remarkably reversed the decreased luciferase activity induced by miR-370 mimics transfection (Fig. 4e). We further investigated the effect of miR-370 on the expression of HMGA2 in A549 and PC9 cells. As expected, HMGA2 expression was decreased in the nucleus of A549 and PC9 cells transfected with miR-370 mimics (Fig. 4g–i), indicating that HMGA2 was a direct target of miR-370.

To validate that circASPH positively regulated HMGA2 by sponging miR-370, we first infected 293T cells with lenti-circASPH or lenti-control to establish stable transfections. Then, the HMGA2 3′-UTR reporter was transiently cotransfected with miR-370 mimics or miR-scramble. We found that luciferase activity, which was evidently abrogated by transduction of miR-370 mimics, was enhanced by circASPH overexpression (Fig. 4f). Taken together, these findings demonstrated that circASPH could upregulate HMGA2 by acting as a sponge for miR-370.

### The tumor-promoting effect of circASPH is mediated by HMGA2

**Fig. 5:**
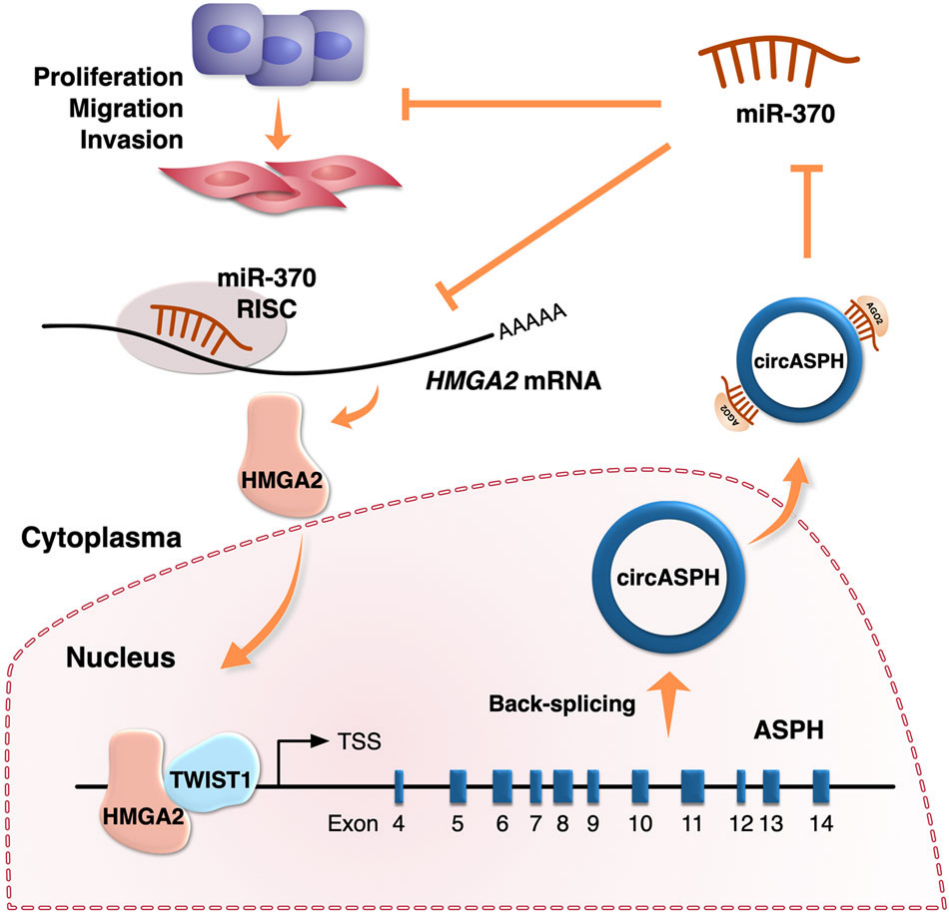
The schematic representation of the whole study. The schematic diagram shows the oncogenic functions of HMGA2-circASPH-HMGA2 axis in lung adenocarcinoma.

According to the aforementioned results, a novel axis (HMGA2-circASPH-HMGA2, mediated by Twist1) was found in A549 and PC9 cells (Fig. 5). To identify the fundamental role of HMGA2 in this axis, a lenti-siHMGA2 vector was constructed to knock down HMGA2 in A549 and PC9 cells, followed by coinfection of lenti-circASPH. We found that lenti-circASPH/siHMGA2 A549 and PC9 cells presented attenuated proliferation, migration, and invasion abilities in vitro (Supplementary Fig. S7a–g). Furthermore, tumor xenograft assays showed decreased tumor weights and volumes in the lenti-circASPH/siHMGA2 group compared with the lenti-circASPH/siControl group (Supplementary Fig. S7h–j).

## Discussion

The functions of HMGA2 have been studied extensively. During the transcription process, HMGA2 alters the structure of DNA and interacts with transcription factors. The important roles of HMGA2 and its downstream genes in tumor progression have been demonstrated in earlier studies^4,5^. Our previous studies have also identified HMGA2 as an oncogene in squamous cell lung carcinoma^6,20^. In this study, using RNA sequencing, we identified specific expression patterns of HMGA2-related circRNAs in the human lung adenocarcinoma cell line A549. Consistent with previous studies^29,30^, we found that most of the HMGA2-related circRNAs were derived from exon circularization. So far, most publications concerning circRNAs have focused on the functions of circRNAs in human diseases. Among these functions, acting as a molecular sponge for miRNAs represents the most conspicuous function^15,22^. However, the regulation of circRNA expression is not well known. According to Meng et al.^21^, circCul2 is directly regulated by Twist1 in hepatocellular carcinoma. In this study, a novel circular RNA, circASPH, originating from the circularization of exon 4 to exon 14 of the ASPH gene, was found to be directly regulated by HMGA2 and Twist1. Along with Meng’s study^21^, our findings improved the understanding of the circRNA regulation network. Furthermore, RNA-editing enzymes, splicing regulators, and RNA-binding proteins have been identified to contribute to the circularization and biogenesis of circRNAs^31–34^. In this study, the RNA-editing enzymes and splicing regulators involved in the circularization of circASPH were not covered. Further relevant experiments are needed to identify the RNA-editing enzymes and splicing regulators, which cooperate with HMGA2 and Twist1 during the biogenesis of circASPH.

Accumulating evidence indicates that circRNAs play important roles in the development of lung cancer^17,35,36^. CircPRKCI has been demonstrated to be sponge for both miR-545 and miR-589, and abrogates their suppression of E2F7 to promote the progression of lung adenocarcinoma^17^. Circular RNA F-circEA-2a is a novel biomarker for lung cancer and contributes to the progression of lung cancer^35^. However, according to Wei et al.^36^, circPTPRA inhibits EMT in lung cancer. Taken together, these findings suggest that circRNAs might play critical but diversified roles in the tumorigenesis of lung cancer. Moreover, circular RNAs are also involved in other malignancies. According to Du et al.^37^, circ-DNMT1 contributes to the progression of breast cancer. In this study, using qRT-PCR assay to examine the tissue samples of lung adenocarcinoma patients, we observed a significantly higher level of circASPH in the cancerous tissue group compared with the adjacent normal tissue group. The correlation between circASPH levels and clinical characteristics was also evaluated. Our data showed that there was a positive correlation between circASPH expression and T stage and N stage. The fact that patients with higher levels of circASPH had a shorter overall survival indicated that circASPH might be a poor prognostic factor. Furthermore, the tumor-promoting effect of circASPH was observed in vitro and in vivo. Our findings, identifying circASPH as an oncogene, support the fact that circRNAs contribute to the carcinogenesis of lung cancer. However, we did not test blood samples from lung adenocarcinoma patients and healthy volunteers. Thus, the baseline circASPH level in healthy individuals and the relationship between tissue and blood levels of circASPH in lung adenocarcinoma patients are unknown. Under this circumstance, further experiments are needed to assess the feasibility of circASPH as a biomarker for lung adenocarcinoma.

The number of studies on the functions and mechanisms of circRNAs in human diseases is gradually increasing. Among the multiple mechanisms identified, circRNAs acting as molecular sponges for miRNAs represent the most conspicuous mechanism^38^, although only a few circRNAs have been found contain multiple binding sites to trap several particular miRNAs^9,29^. However, according to Zhang et al.^39^, in addition to mRNA, circRNA also encodes protein, and the protein product of circSHPRH suppresses glioma tumorigenesis. Circ-ZNF609 is also an example of a protein-coding circRNA in eukaryotes^12^. Furthermore, the interaction between circRNA and protein and the function of the circRNA–protein complex were demonstrated by Du et al.^40^. Herein, we showed that circASPH contained binding sites for multiple miRNAs and captured miR-370 in A549 and PC9 cells. Our findings provide an example of a circRNA functioning as a molecular sponge for miRNAs in lung adenocarcinoma.

Although most circRNAs are noncoding RNAs, they still influence gene expression at the protein level via several mechanisms. Circ-ITCH was found to modulate the miR-17 (or miR-224) targeting of p21 and PTEN^15^. Interestingly, Li et al. found that circITGA7 enhanced expression of its host gene ITGA7 via the Ras pathway^27^. In this study, we observed the positive regulation of HMGA2 by circASPH in vitro and in vivo. Mechanistically, circASPH was identified to act as a molecular sponge for miR-370 and abrogate miR-370-mediated inhibition of HMGA2.

Overall, this study revealed the oncogenic functions of the HMGA2-circASPH-HMGA2 axis in lung adenocarcinoma and may be useful in developing circRNA-based therapeutic strategies for lung adenocarcinoma.

## Supplementary information

Supplementary Figure S1

Supplementary Figure S2

Supplementary Figure S3

Supplementary Figure S4

Supplementary Figure S5

Supplementary Figure S6

Supplementary Figure S7

Supplementary File 1

Supplementary File 2

Supplementary Table 1

Supplementary Table 2

## References

[1] Mayr, C., Hemann, M. T. & Bartel, D. P. Disrupting the pairing between let-7 and Hmga2 enhances oncogenic transformation. *Science* **315**, 1576–1579 (2007). 17322030 10.1126/science.1137999 PMC2556962

[2] Yang, E. et al. Dysregulated protease activated receptor 1 (PAR1) promotes metastatic phenotype in breast cancer through HMGA2. *Oncogene* **35**, 1529–1540 (2016). 26165842 10.1038/onc.2015.217 PMC6818098

[3] Chen, X. et al. P53-induced miR-1249 inhibits tumor growth, metastasis, and angiogenesis by targeting VEGFA and HMGA2. *Cell Death Dis.* **10**, 131 (2019). 30755600 10.1038/s41419-018-1188-3 PMC6372610

[4] Sun, M. et al. HMGA2/TET1/HOXA9 signaling pathway regulates breast cancer growth and metastasis. *Proc. Natl Acad. Sci. USA* **110**, 9920–9925 (2013). 23716660 10.1073/pnas.1305172110 PMC3683728

[5] Li, Y. et al. HMGA2 induces transcription factor Slug expression to promote epithelial-to-mesenchymal transition and contributes to colon cancer progression. *Cancer Lett.* **355**, 130–140 (2014). 25218351 10.1016/j.canlet.2014.09.007

[6] Xu, L. et al. miR-541 suppresses proliferation and invasion of squamous cell lung carcinoma cell lines via directly targeting high‐mobility group AT‐hook 2. *Cancer Med.* **7**, 2581–2591 (2018). 29659195 10.1002/cam4.1491 PMC6010725

[7] Jeck, W. R. & Sharpless, N. E. Detecting and characterizing circular RNAs. *Nat. Biotechnol.* **32**, 453–461 (2014). 24811520 10.1038/nbt.2890 PMC4121655

[8] Li, Y. et al. Circular RNA is enriched and stable in exosomes: a promising biomarker for cancer diagnosis. *Cell Res.* **25**, 981–984 (2015). 26138677 10.1038/cr.2015.82 PMC4528056

[9] Hansen, T. B. et al. Natural RNA circles function as efficient microRNA sponges. *Nature* **495**, 384–388 (2013). 23446346 10.1038/nature11993

[10] Memczak, S. et al. Circular RNAs are a large class of animal RNAs with regulatory potency. *Nature* **495**, 333–338 (2013). 23446348 10.1038/nature11928

[11] Pamudurti, N. R. et al. Translation of circRNAs. *Mol. Cell* **66**, 9–21.e7 (2017). 28344080 10.1016/j.molcel.2017.02.021 PMC5387669

[12] Legnini, I. et al. Circ-ZNF609 is a circular RNA that can be translated and functions in myogenesis. *Mol. Cell* **66**, 22–37 (2017). 28344082 10.1016/j.molcel.2017.02.017 PMC5387670

[13] Liu, Z. et al. CircRNA-5692 inhibits the progression of hepatocellular carcinoma by sponging miR-328-5p to enhance DAB2IP expression. *Cell Death Dis.* **10**, 900 (2019). 31776329 10.1038/s41419-019-2089-9 PMC6881381

[14] Yan, D. et al. Circular RNA circPICALM sponges miR-1265 to inhibit bladder cancer metastasis and influence FAK phosphorylation. *EBioMedicine* **48**, 316–331 (2019). 31648990 10.1016/j.ebiom.2019.08.074 PMC6838432

[15] Yang, C. et al. Circular RNA circ-ITCH inhibits bladder cancer progression by sponging miR-17/miR-224 and regulating p21, PTEN expression. *Mol. Cancer* **17**, 19 (2018). 29386015 10.1186/s12943-018-0771-7 PMC5793418

[16] Chen, B. et al. circEPSTI1 as a prognostic marker and mediator of triple-negative breast cancer progression. *Theranostics* **8**, 4003–4015 (2018). 30083277 10.7150/thno.24106 PMC6071524

[17] Qiu, M. et al. The circular RNA circPRKCI promotes tumor growth in lung adenocarcinoma. *Cancer Res.* **78**, 2839–2851 (2018). 29588350 10.1158/0008-5472.CAN-17-2808

[18] Bray, F. et al. Global cancer statistics 2018: GLOBOCAN estimates of incidence and mortality worldwide for 36 cancers in 185 countries. *CA Cancer J. Clin.* **68**, 394–424 (2018). 30207593 10.3322/caac.21492

[19] Zhang, X. W. et al. Twist-related protein 1 negatively regulated osteoblastic transdifferentiation of human aortic valve interstitial cells by directly inhibiting runt-related transcription factor 2. *J. Thorac. Cardiovasc. Surg.* **148**, 1700–1708 (2014). 24703637 10.1016/j.jtcvs.2014.02.084

[20] Xu, L. et al. ANG promotes proliferation and invasion of the cell of lung squamous carcinoma by directly up-regulating HMGA2. *J Cancer.* **7**, 862–871 (2016). 27162546 10.7150/jca.14440 PMC4860804

[21] Meng, J. et al. Twist1 regulates vimentin through Cul2 circular RNA to promote EMT in hepatocellular carcinoma. *Cancer Res.* **78**, 4150–4162 (2018). 29844124 10.1158/0008-5472.CAN-17-3009

[22] Wang, R. et al. CircNT5E acts as a sponge of miR-422a to promote glioblastoma tumorigenesis. *Cancer Res.* **78**, 4812–4825 (2018). 29967262 10.1158/0008-5472.CAN-18-0532

[23] Bulatov, E. et al. Isatin-Schiff base-copper (II) complex induces cell death in p53-positive tumors. *Cell Death Discov.* **4**, 103 (2018). 30455989 10.1038/s41420-018-0120-z PMC6234212

[24] Gou, W. et al. CD74-ROS1 G2032R mutation transcriptionally up-regulates Twist1 in non-small cell lung cancer cells leading to increased migration, invasion, and resistance to crizotinib. *Cancer Lett.* **422**, 19–28 (2018). 29477381 10.1016/j.canlet.2018.02.032

[25] Hwang, W. et al. Expression of neuroendocrine factor VGF in lung cancer cells confers resistance to EGFR kinase inhibitors and triggers epithelial-to-mesenchymal transition. *Cancer Res.* **77**, 3013–3026 (2017). 28381546 10.1158/0008-5472.CAN-16-3168

[26] Huang, X. et al. Wnt7a activates canonical Wnt signaling, promotes bladder cancer cell invasion, and is suppressed by miR-370-3p. *J. Biol. Chem.* **293**, 6693–6706 (2018). 29549123 10.1074/jbc.RA118.001689 PMC5936804

[27] Li, X. et al. Circular RNA circITGA7 inhibits colorectal cancer growth and metastasis by modulating the Ras pathway and upregulating transcription of its host gene ITGA7. *J. Pathol.* **246**, 166–179 (2018). 29943828 10.1002/path.5125

[28] Du, F. et al. miR-137 alleviates doxorubicin resistance in breast cancer through inhibition of epithelial-mesenchymal transition by targeting DUSP4. *Cell Death Dis.* **10**, 922 (2019). 31801953 10.1038/s41419-019-2164-2 PMC6892819

[29] Zheng, Q. et al. Circular RNA profiling reveals an abundant circHIPK3 that regulates cell growth by sponging multiple miRNAs. *Nat. Commun.* **7**, 11215 (2016). 27050392 10.1038/ncomms11215 PMC4823868

[30] Yang, Y. et al. Novel role of FBXW7 circular RNA in repressing glioma tumorigenesis. *J. Natl Cancer Inst*. **110**, 304–315 (2018). 10.1093/jnci/djx166 PMC6019044 28903484

[31] Aktaş, T. et al. DHX9 suppresses RNA processing defects originating from the Alu invasion of the human genome. *Nature* **544**, 115–119 (2017). 28355180 10.1038/nature21715

[32] Liang, D. et al. The output of protein-coding genes shifts to circular RNAs when the pre-mRNA processing machinery is limiting. *Mol. Cell* **68**, 940–954 (2017). 29174924 10.1016/j.molcel.2017.10.034 PMC5728686

[33] Conn, S. J. et al. The RNA binding protein quaking regulates formation of circRNAs. *Cell* **160**, 1125–1134 (2015). 25768908 10.1016/j.cell.2015.02.014

[34] Khan, M. A. et al. RBM20 regulates circular RNA production from the titin gene. *Circ. Res.* **119**, 996–1003 (2016). 27531932 10.1161/CIRCRESAHA.116.309568

[35] Tan, S. et al. Circular RNA F-circEA-2a derived from EML4-ALK fusion gene promotes cell migration and invasion in non-small cell lung cancer. *Mol. Cancer* **17**, 138 (2018). 30236141 10.1186/s12943-018-0887-9 PMC6146612

[36] Wei, S. et al. The circRNA circPTPRA suppresses epithelial-mesenchymal transitioning and metastasis of NSCLC cells by sponging miR-96-5p. *EBioMedicine* **44**, 182–193 (2019). 31160270 10.1016/j.ebiom.2019.05.032 PMC6604667

[37] Du, W. W. et al. A circular RNA circ-DNMT1 enhances breast cancer progression by activating autophagy. *Oncogene* **37**, 5829–5842 (2018). 29973691 10.1038/s41388-018-0369-y

[38] Wu, J. et al. CircIRAK3 sponges miR-3607 to facilitate breast cancer metastasis. *Cancer Lett.* **430**, 179–192 (2018). 29803789 10.1016/j.canlet.2018.05.033

[39] Zhang, M. et al. A novel protein encoded by the circular form of the SHPRH gene suppresses glioma tumorigenesis. *Oncogene* **37**, 1805–1814 (2018). 29343848 10.1038/s41388-017-0019-9

[40] Du, W. W. et al. Foxo3 circular RNA retards cell cycle progression via forming ternary complexes with p21 and CDK2. *Nucleic Acids Res.* **44**, 2846–2858 (2016). 26861625 10.1093/nar/gkw027 PMC4824104

