# Supplementary figures and images for: HMGA2 regulates circular RNA ASPH to promote tumor growth in lung adenocarcinoma
Source: Cell Death Dis. 2020 Jul 27;11(7):593. doi: 10.1038/s41419-020-2726-3 (PMC7385491; doi:10.1038/s41419-020-2726-3)

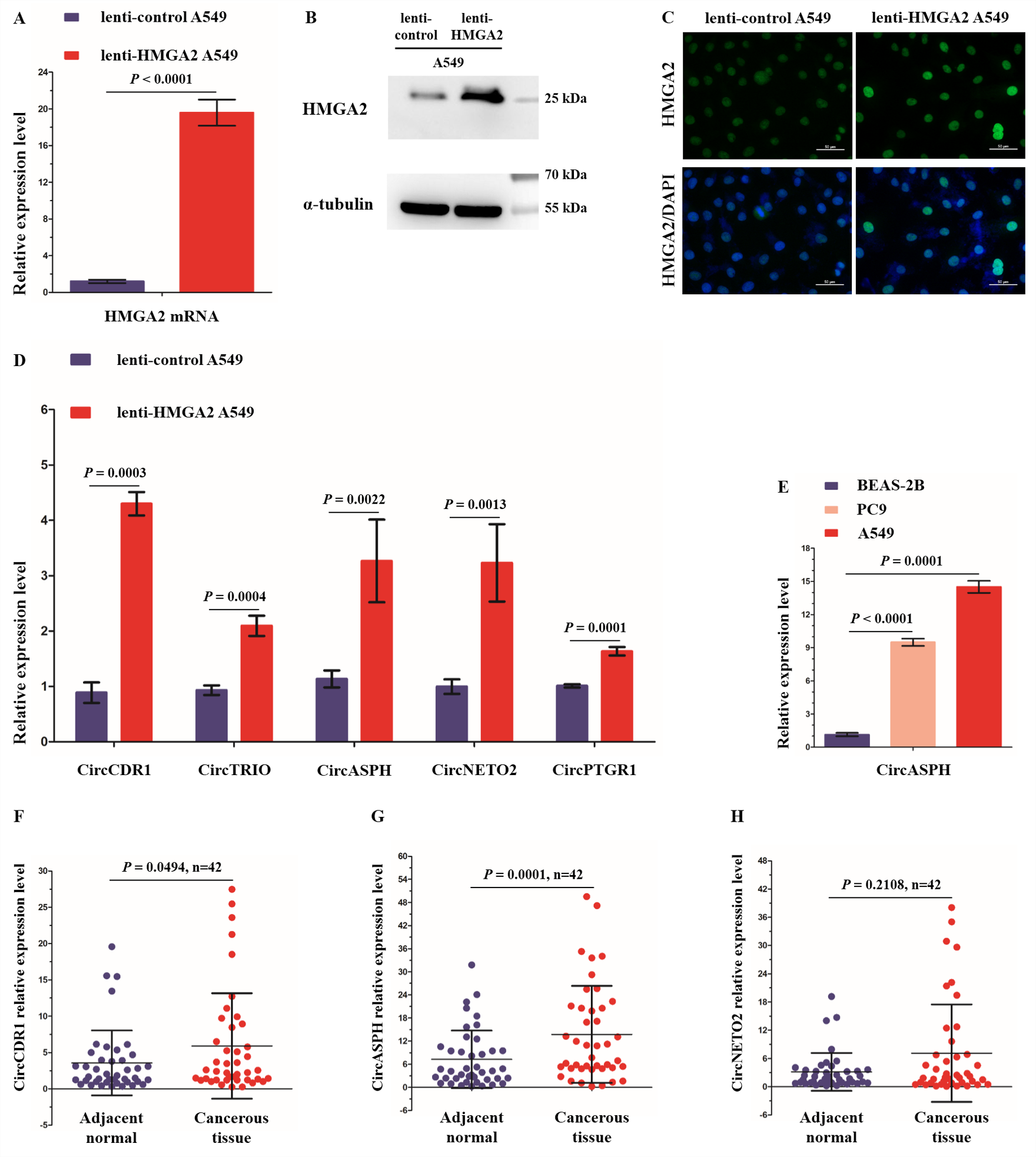

Supplement: Supplementary file 1 — Supplementary Figure S1 [file 41419_2020_2726_MOESM1_ESM.tif]

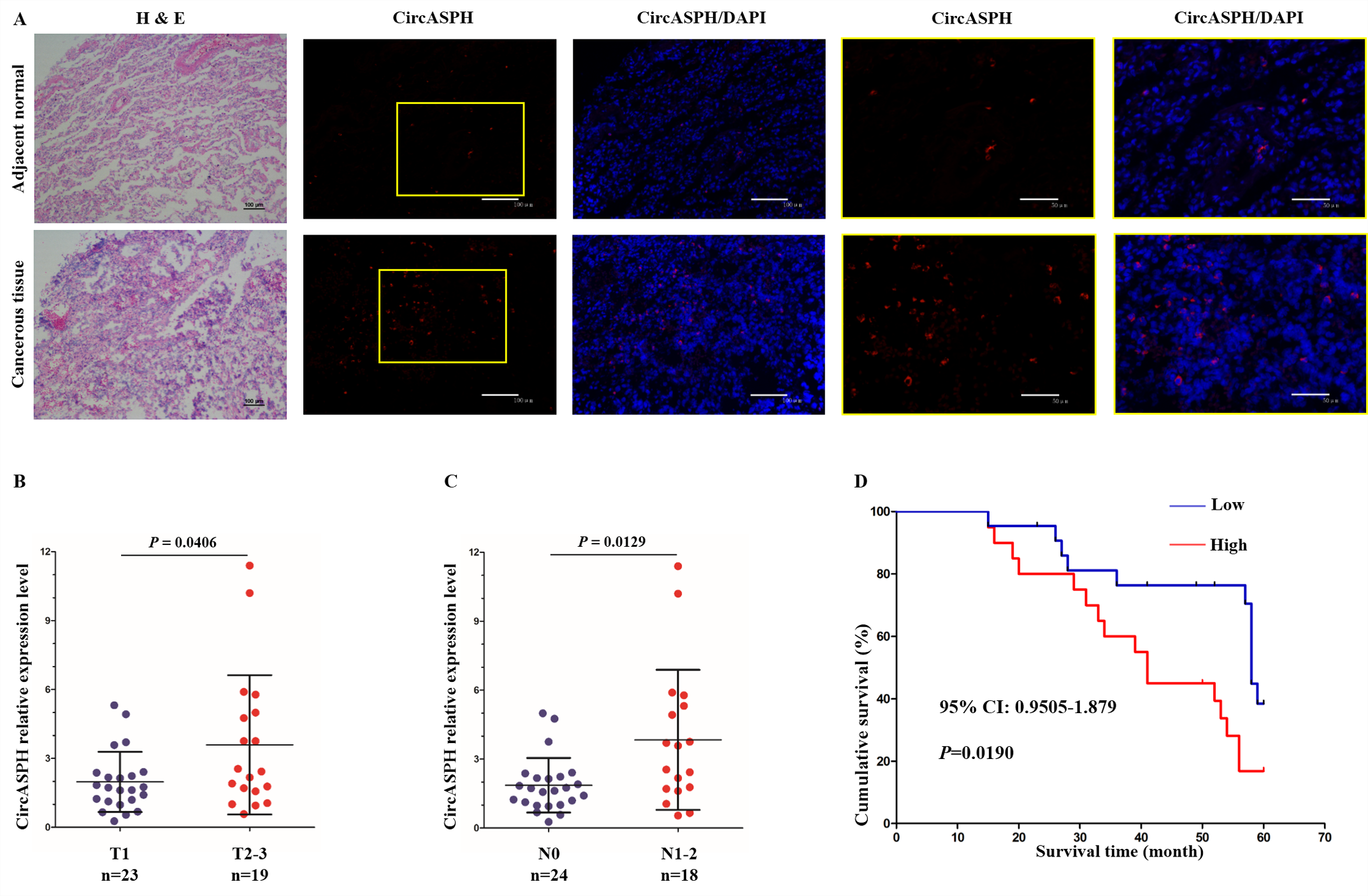

Supplement: Supplementary file 2 — Supplementary Figure S2 [file 41419_2020_2726_MOESM2_ESM.tif]

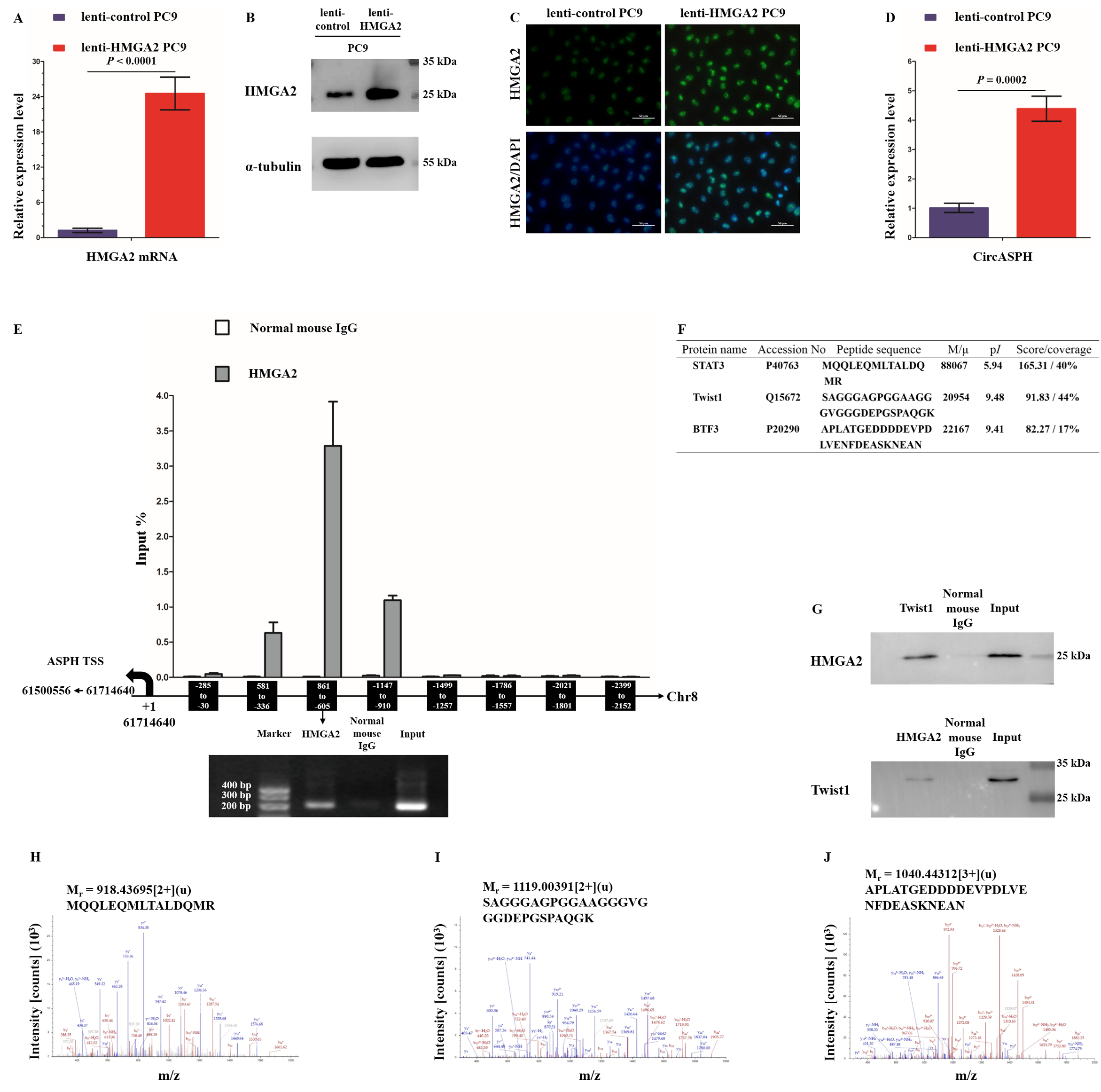

Supplement: Supplementary file 3 — Supplementary Figure S3 [file 41419_2020_2726_MOESM3_ESM.tif]

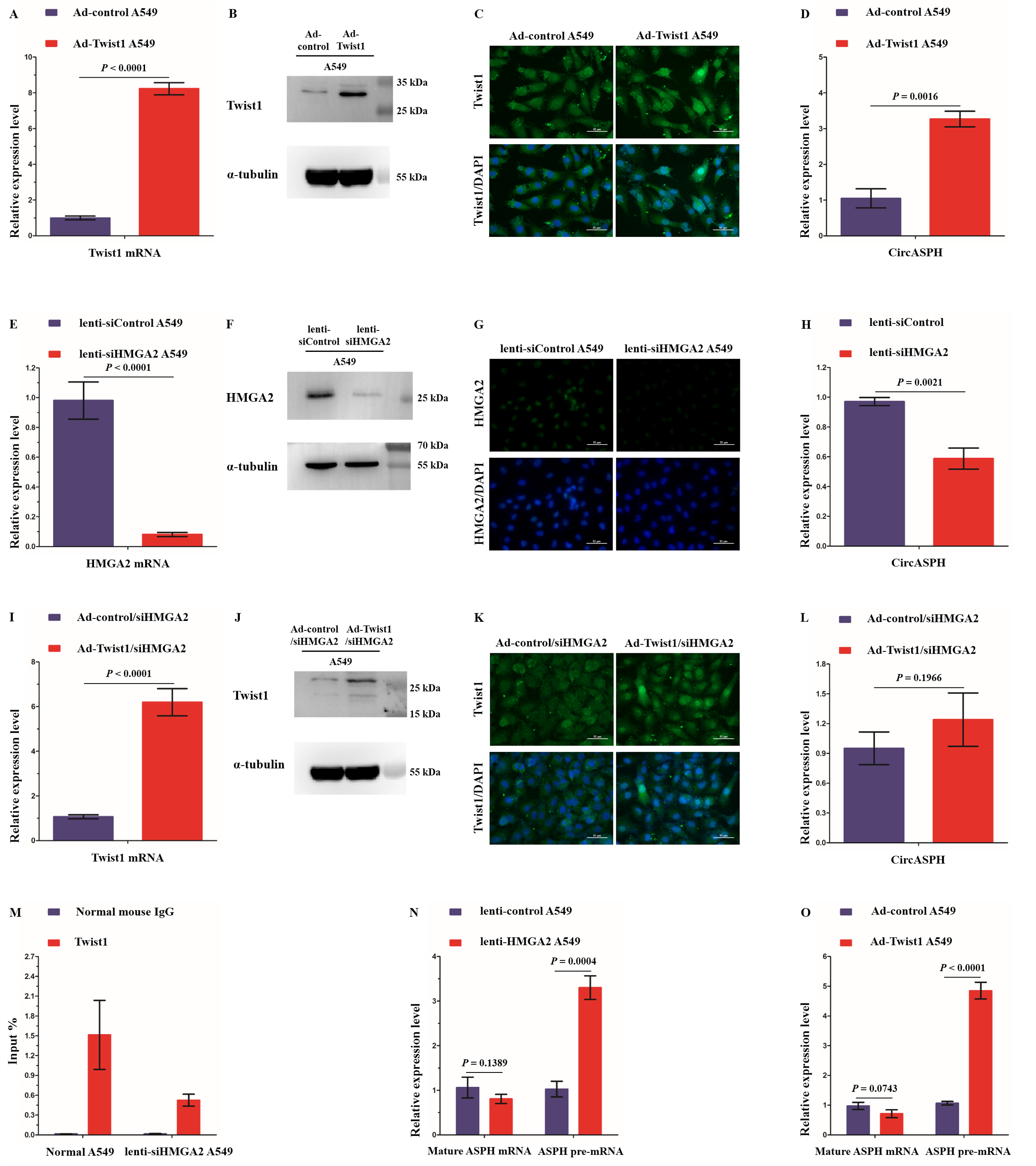

Supplement: Supplementary file 4 — Supplementary Figure S4 [file 41419_2020_2726_MOESM4_ESM.tif]

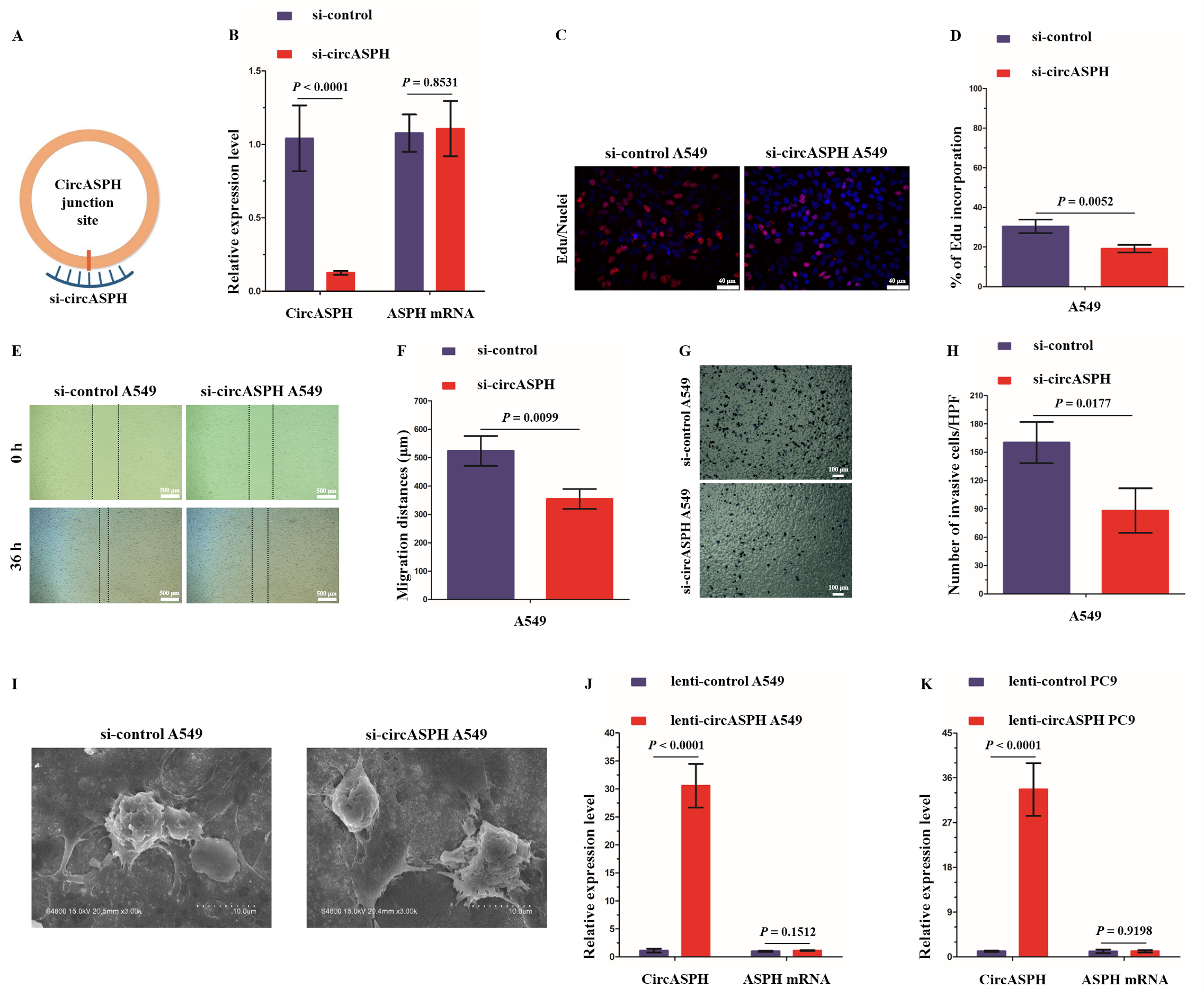

Supplement: Supplementary file 5 — Supplementary Figure S5 [file 41419_2020_2726_MOESM5_ESM.tif]

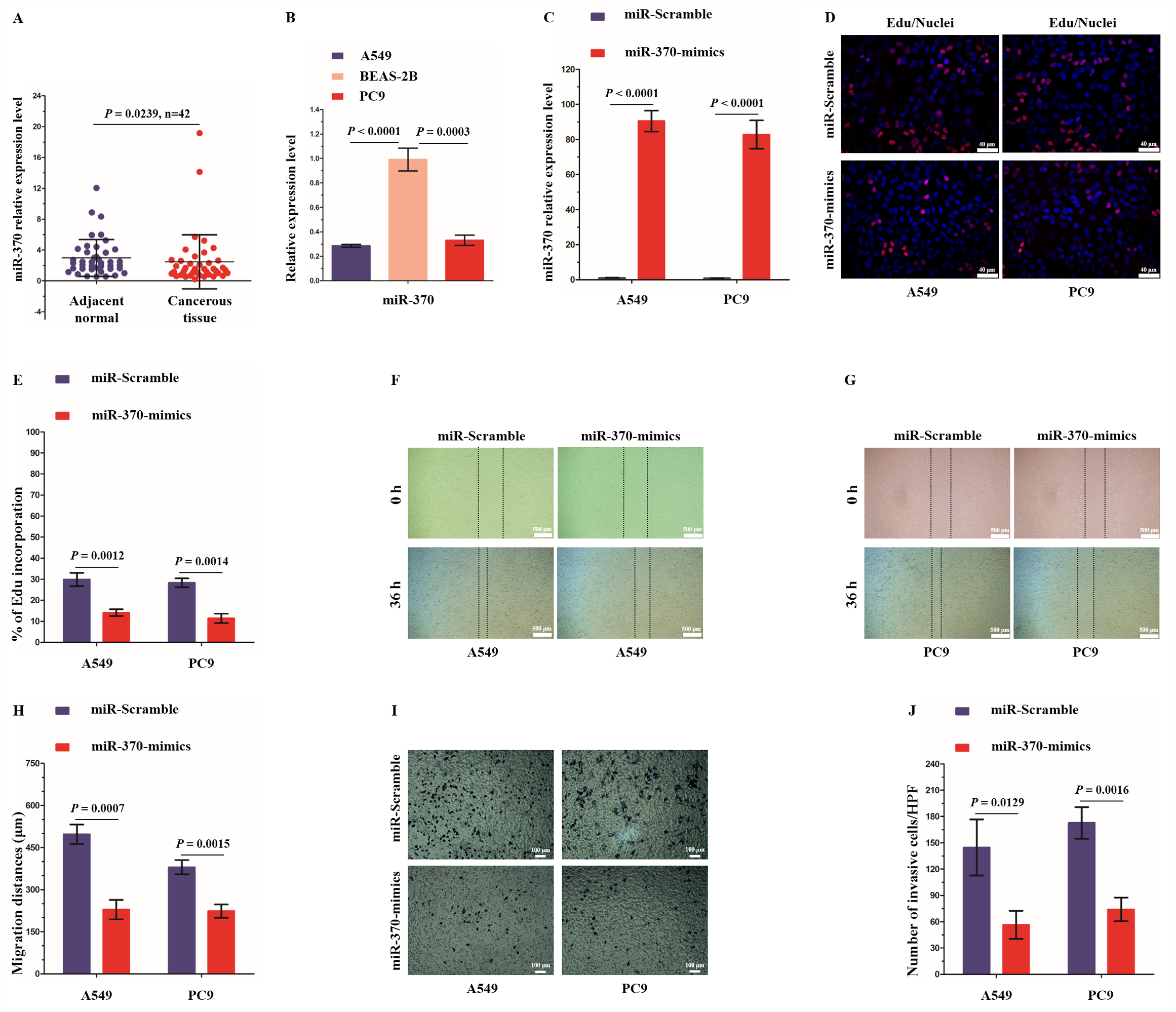

Supplement: Supplementary file 6 — Supplementary Figure S6 [file 41419_2020_2726_MOESM6_ESM.tif]

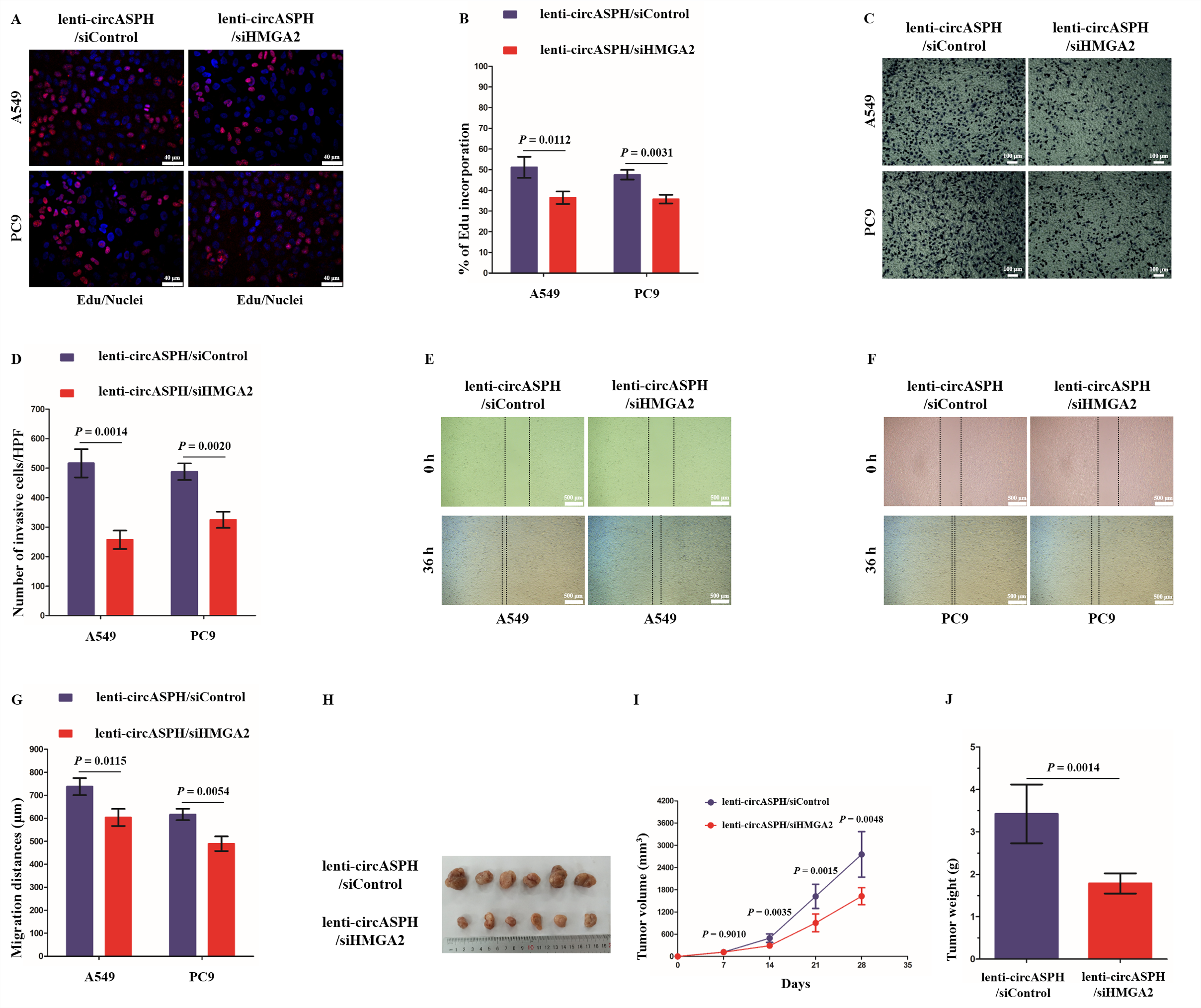

Supplement: Supplementary file 7 — Supplementary Figure S7 [file 41419_2020_2726_MOESM7_ESM.tif]
