# Supplementary material for: HMGA2 regulates circular RNA ASPH to promote tumor growth in lung adenocarcinoma
Source: Cell Death Dis. 2020 Jul 27;11(7):593. doi: 10.1038/s41419-020-2726-3 (PMC7385491; doi:10.1038/s41419-020-2726-3)
Supplement: Supplementary file 8 — Supplementary File 1 [file 41419_2020_2726_MOESM8_ESM.docx]

**Cell culture**

The human lung adenocarcinoma cell lines A549 and PC9 and human bronchial epithelial cell line BEAS-2B were obtained from Shanghai Cell Bank, Shanghai, China and were cultured in DMEM supplemented with 10% fetal bovine serum (FBS), 100 μg/mL streptomycin, and 100 units/mL penicillin at 37 °C in a 5% CO_2_ atmosphere.

**HMGA2, Twist1, and circASPH overexpression**

A lentivirus vector expressing HMGA2 (lenti-HMGA2, GOCL3651094131) and a control lentivirus vector (lenti-control) were constructed by GeneChem Co., Ltd. (Shanghai, China). An adenovirus vector expressing Twist1 (Ad-Twist1, GOCA3451039423) and its control vector (Ad-control), and a lentivirus vector expressing circASPH (lenti-circASPH) and its control vector (lenti-control) were constructed by OBiO Technology Corp., Ltd. (Shanghai, China).

**MicroRNA overexpression**

The miR-1182-mimics, miR-1236-mimics, miR-370-mimics, miR-375-mimics, and miR-scramble were synthesized by GenePharma (Shanghai, China). After the cells entered the logarithmic phase, the miR-mimics and miR-scramble transfections were conducted according to the Lipofectamine RNAiMAX (13778030; Invitrogen) instruction. The sequences of miR-scramble were as follows: sense strand: 5’-UUCUCCGAACGUGUCACGUTT-3’; and anti-sense strand: 5’-ACGUGACACGUUCGGAGAATT-3’.

**RNase R treatment**

Total RNA from the whole-cell lysates were isolated using TRIzol reagent (15596026; Invitrogen) according to the manufacturer’s instructions. 2 μg of the total RNA was incubated for 20 min at 37 °C with or without 3U/μg RNase R (RNR07250; Epicentre Technologies). The resulting RNA was purified with a RNeasy MinElute Cleanup Kit (74204; QIAGEN) according to the manufacturer’s protocol.

**RNA sequencing (RNA-seq) analysis**

In brief, after the stable overexpression of HMGA2 in A549 cells was confirmed, total RNA from the lenti-HMGA2 and lenti-control A549 cells were isolated using TRIzol reagent (15596026; Invitrogen). Then the RNA samples were treated with RNase R to remove other non-circular RNAs and purified. The treated RNA of each cell group (n = 3 for each group, totally 6 samples) was used to prepare the circRNA sequencing library, which included the following steps: a) 2 μg treated RNA were pretreated to enrich circRNA and remove ribosomal RNA (rRNA) using CircRNA Enrichment Kit (Cloud-seq Inc, USA); b) RNA libraries were constructed by using pretreated RNAs with TruSeq Stranded Total RNA Library Prep Kit (RS-122-2201; Illumina, San Diego, CA, USA) according to the manufacturer’s instructions; c) Libraries were controlled for quality and quantified using the BioAnalyzer 2100 system (Agilent Technologies, Inc., USA). The libraries were denatured as single-stranded DNA molecules, captured on Illumina flow cells, amplified in situ as clusters and finally sequenced for 150 cycles on Illumina HiSeq Sequencer according to the manufacturer’s instructions. Paired-end reads were harvested from Illumina HiSeq 4000 sequencer and were quality-controlled by Q30. After 3’ adaptor-trimming and low-quality reads removed by Cutadapt software (v1.9.3), the reads were aligned to the reference genome/transcriptome with STAR software, and circRNAs were detected and annotated with DCC software. CircBase database and circ2Trait disease database were used to annotate the identified circRNAs. The junction read counts were normalized, and the differentially expressed circRNAs were determined using edgeR package of R software.

**Quantitative real-time reverse-transcription polymerase chain reaction (qRT-PCR)**

qRT-PCR assays were performed as described elsewhere [6]. β-actin and U6 snRNA served as the internal controls. The 2^-ΔΔCT^ method was used to process the raw data and to determine the relative expression levels. To statistically compare the differences between groups, log_2_ transformation was applied to analyze the relative expression results and to obtain the normally distributed data. The primers used in the qRT-PCR assays are as follows: HMGA2 forward 5’-AGCAGCAGCAAGAACCAACC-3’ and reverse 5’-CCTGAGCAGGCTTCTTCTGA-3’; Twist1 forward 5’- GTCCGCAGTCTTACGAGGAG-3’ and reverse 5’- TGGAGGACCTGGTAGAGGAA-3’; β-actin forward 5’-CCTGGCACCCAGCACAAT-3’ and reverse 5’-GGGCCGGACTCGTCATAC-3’; circCDR1 forward 5’- TTCCAACGTCTCCAGTGTGCT-3’ and reverse 5’- AGGTGCCATCGGAAACCCTG-3’; circTRIO forward 5’- TTCCACCAGAAGGCCGAAAA-3’ and reverse 5’- GACCATATTTGTCTCAGAATAAGCA-3’; circASPH forward 5’-ACTGCTCCCCCTGAGGAT-3’ and reverse 5’-GGGACTGCTGGCTCTGAA-3’; circNETO2 forward 5’-GAGCTTGAAGGACTGGGATT-3’ and reverse 5’- GGCGAAGCAAAATGACCTCC-3’; circPTGR1 forward 5’-ACCATCAGGAGACGCTTTCT-3’ and reverse 5’-TTACCTCACGCAACAGGTCG-3’; ASPH1 forward 5’-GGGTGAAGTCTTCCCAGGTG-3’ and reverse 5’-CCGCCTTTCCTCCCATTCTT-3’; ASPH2 forward 5’-CCCAGCGTAAGAATGCCAAG-3’ and reverse 5’-GGTGGGACCTGGGGAGAT-3’; miR-370 forward 5’-TGTAACCAGAGAGCGGGATGT-3’ and reverse 5’-TTTTGGCATAACTAAGGCCGAA-3’; U6 snRNA forward 5’-CTCGCTTCGGCAGCACATATACT-3’ and revers 5’-ACGCTTCACGAATTTGCGTGTC-3’. ASPH1 was designed to amplify the mature mRNA (exon 1 and exon 2) of ASPH. ASPH2 was designed to amplify the pre-mRNA of ASPH (exon 1 and intron 1).

**Western blot**

Western blotting was performed as described elsewhere [6]. The primary antibodies HMGA2 (1:5000, PA5-21320; Invitrogen), Twist1 (1:500, 25465-1-AP; Proteintech), and α-tubulin (1:200, BM3885; BOSTER) were used, with α-tubulin serving as the internal control. Peroxidase-AffiniPure goat anti-rabbit IgG (H+L) (1:5000, 111035003; Jackson ImmunoResearch) was used as the secondary antibody.

**Immunocytochemistry (ICC)**

Immunocytochemistry was performed as described elsewhere [6]. The primary antibodies used were anti-HMGA2 (1:500, PA5-21320; Invitrogen) and anti-Twist1 (1:100, 25465-1-AP; Proteintech).

**RNA interference**

CircASPH was specifically knockdown using the siRNA (si-circASPH: 5’-GCAAAAGGACUUUAAAGAGAUU-3’) to target the back-splice junction of circASPH. The negative control siRNA (si-control, AM4637) was purchased from Invitrogen. The transfections were conducted under the instructions of Lipofectamine RNAiMAX (13778030; Invitrogen). 36 hours after transfection, A549 cells were harvested for qRT-PCR analysis of circASPH and ASPH mature mRNA. A lentivirus vector expressing siRNA to specifically knock down HMGA2 (lenti-siHMGA2) and a control vector (lenti-siControl) were constructed by GenePharma (Shanghai, China). The sequence of siHMGA2 was: sense strand1 5’-CCAGGAAGCAGCAGCAAGAUU-3’ and sense strand2 5’-CGGCCAAGAGGCAGACCUAUU-3’.

**RNA-binding protein immunoprecipitation (RIP)**

The Ago2-RIP assay was performed in A549 and PC9 cells, using EZ-Magna RIP^TM^ RNA-Binding Protein Immunoprecipitation Kit (17-701; Merck Millipore) according to the manufacturer’s instructions. The immunoprecipitating antibody was anti-Argonaute-2 (Ago2) antibody (ab32381; Abcam). For the negative control, normal mouse IgG in the kit was used. The immunoprecipitated RNAs was extracted by RNeasy MinElute Cleanup Kit (74204; QIAGEN) according to the instructions. The abundance of circASPH was evaluated by RT-PCR.

**Biotin-coupled miRNA capture**

A549 and PC9 cells were transfected with biotinylated miR-370 mimics or miR-scramble (GenePharma, China) according to the standard protocol of Lipofectamine^TM^ 3000 (L3000015; Invitrogen). 36 hours after transfection, cells were harvested, washed in cold PBS, and lysed in lysis buffer. 50 μL streptavidin-conjugated magnetic beads were activated and blocked with blocking buffer for 2 h. Then the beads were incubated with cell lysates at 4 °C for 8 h to pull down the biotin-coupled RNA complex. Lysis buffer was used to wash beads. TRIzol LS (10296028; Invitrogen) was used to extract RNAs specifically interacting with miR-370. The abundance of circASPH was evaluated by qRT-PCR and semi-qPCR.

**Cell proliferation**

For EdU (5-ethynyl-2’-deoxyuridine) assay, a Yefluor 594 EdU Imaging Kit with Yefluor 594 Azied (40276ES60; YEASEN) was used according to the manufacturer’s instructions. Nucleic acids were stained with Hoechst 33342.

**Chromatin immunoprecipitation (ChIP)**

The chromatin immunoprecipitation assay was performed using the Low Cell ChIP-Seq Kit (53084; Active Motif) and the ChIP-IT Control Kit (53010; Active Motif) according to the manufacturer’s instructions. After entering the logarithmic phase, the A549 cells were collected and lysed. The immunoprecipitating antibodies were anti-HMGA2 antibody (PA5-21320; Invitrogen) and anti-Twist antibody (25465-1-AP; Proteintech). The mouse IgG in the Control Kit was used as negative control. The sequences of primers used were as follows: ASPH1 forward 5’- AGTGCAGAAGGGAAATGTGG-3’ and reverse 5’- CTGATGCAAGAGGTGGGTTC-3’ (-30 to -285); ASPH2 forward 5’- AAAATTTGACTGCCCCACTC-3’ and reverse 5’-GTCCCCCAAAGCCTTAACTC-3’ (-336 to -581); ASPH3 forward 5’-TGAAATGTGAGGGCATGAAA-3’ and reverse 5’-GGTGGCAAGAGAAAATGAGG-3’ (-605 to -861); ASPH4 forward 5’-CCAGCCATGTGGAACTGTAA-3’ and reverse 5’- TTCAAAACACAATGCTCTAGCC-3’ (-910 to -1147); ASPH5 forward 5’-CCCTGAATGGGAGAAAGGAT-3’ and reverse 5’- CCTTTTCACCTGTGGATGGT-3’ (-1257 to -1499); ASPH6 forward 5’- GGCTGGAAAAGAGGAATGAA-3’ and reverse 5’- CTTGAGATCAGCGACGTCTT-3’ (-1557 to -1786); ASPH7 forward 5’- AACCACGTGCCAAGACATTT-3’ and reverse 5’-TTCATCCATTTGGTGCTGAA-3’ (-1801 to -2021); ASPH8 forward 5’-GAAAGGGGACAAAATCCACA-3’ and reverse 5’-CCTGCTCACTTAGGGCTGTT-3’ (-2152 to -2399). For RT-PCR, the amplification difference between the negative control and HMGA2 or Twist1 varying from 3 to 12 cycles was considered significant. For semi-qPCR, amplifications were performed with 30 cycles in a total volume of 20 μL, and the PCR products were subjected to a 4% agarose gel for electrophoresis.

**Co-IP and protein mass spectrometry assay**

The Co-IP assay was performed using A549 cell lysates and Pierce^TM^ Co-Immunoprecipitation Kit (26149; Thermo Scientific). The immunoprecipitating antibody was anti-HMGA2 antibody (PA5-21320; Invitrogen) and anti-Twist1 antibody (25465-1-AP; Proteintech). For the negative control, normal mouse IgG (ab188776; Abcam) was used. One part of the IP eluate was measured with liquid chromatography with tandem mass spectrometry. Peptides were identified using Sequest HT (Thermo Scientific) through Protein Discoverer, version 1.17. MS/MS data were searched using 10ppm mass accuracy on precursor m/z and a 0.5Da window on fragment ions. Fully enzymatic tryptic searches with up to three missed cleavage sites were allowed. Oxidized methionines were searched as a variable modification and alkylated cysteines were searched as a fixed modification. Sequential database searches were performed using the NCBI RefSeqHuman FASTA database. Peptides for each charge state were filtered to a false discovery rate (FDR) of 1%. The other part of the IP eluate was prepared for western blot assay.

**Scanning electron microscopy assay**

After fixed in 2.5% glutaric dialdehyde for 4 hours, the cells were rinsed with PBS for 3 times. Then, the cells were re-fixed with 1% osmic acid for 2.5 hours and rinsed with PBS for 3 times again. After dehydrated in a gradient concentration of ethyl alcohol, cells were dried using CO_2_ critical point drying method. Then, the cells were coated with gold and photographed. The type of scanning electron microscopy is S-4800 (Hitachi, Japan).

**Luciferase assay**

Lipofectamine 2000 (Invitrogen) was used to cotransfect 293T cells in the following groups: 1) psiCHECK-2-circASPH and miR-Scramble VS psiCHECK-2-circASPH and miR-mimics; 2) psiCHECK-2-HMGA2-WT and miR-Scramble VS psiCHECK-2-HMGA2-WT and miR-370 mimics; 3) psiCHECK-2-HMGA2-WT and miR-370 mimics VS psiCHECK-2-HMGA2-MU and miR-370 mimics. The luciferase activity was measured using the Promega Dual-Luciferase system. The psiCHECK-2-circASPH and psiCHECK-2-HMGA2-WT plasmids containing the whole sequence of circASPH and the HMGA2 mRNA 3’ UTRs respectively, were constructed by PCR cloning of chemically synthesized DNA fragments, using the following primers: psiCHECK-2-circASPH forward 5’-CGCTCGAGGACTTAAAGAGAGATCTACT-3’ and reverse 5’-AGCGGCCGCCTTTTGCTTTTTGTTCTGGA-3’; psiCHECK-2-HMGA2-WT forward 5’-CGCTCGAGGGGGCGCCAACGTTCGATTTC-3’ and reverse 5’-AGCGGCCGCTCTGTTTTGACCAAACTTTAT-3’. The underlined sequences indicate the restriction enzyme sites for Xhol and Notl, respectively. The psiCHECK-2-HMGA2-MU plasmid containing site-directed mutations was generated using the following primers: forward 5’-TGGATATCACACATATCCTACTTAGTAATAA-3’ and reverse 5’-AGATAAATACCTATAGTGTGTATAGGATGAA-3’. The underlined sequences indicate the mutated sites.

**Xenografts in mice**

The A549 cells used in animal studies experiments were divided into the following groups: 1) lenti-circASPH VS lenti-control; 2) siHMGA2/lenti-circASPH VS siControl/lenti-circASPH. About 5×10^7^ cells were subcutaneously injected into the axilla of female athymic BALB/c nude mice (5 weeks old, 6 mice/group). The growth of tumors was measured once a week and average tumor volume (TV) was calculated according to the formula: TV = (W^2^× L)/2 (W: tumor width; L: tumor length). All mice were sacrificed at 4 weeks post injection, after which tumors were removed and prepared for the western blot and IHC assay. The animal studies were performed in accordance with the institutional ethics guidelines for animal experiments and were approved by the Institutional Review Boards of General Hospital of Western Theater Command.
