# Supplementary material for: HMGA2 regulates circular RNA ASPH to promote tumor growth in lung adenocarcinoma
Source: Cell Death Dis. 2020 Jul 27;11(7):593. doi: 10.1038/s41419-020-2726-3 (PMC7385491; doi:10.1038/s41419-020-2726-3)
Supplement: Supplementary file 9 — Supplementary File 2 [file 41419_2020_2726_MOESM9_ESM.docx]

**Supplementary Figure legends**

**Fig. S1 Verification of the overexpression of HMGA2 and the expression levels of candidate circRNAs.** **a-c** qRT-PCR, western blot, and immunocytochemistry (ICC) analysis of the expression levels of HMGA2 in the HMGA2-overexpressing and control A549 cells. α-tubulin served as the internal control. **d** qRT-PCR analysis of the levels of the 5 candidate circRNAs in HMGA2-overexpressing and control A549 cells. **e** CircASPH expression levels were higher in A549 and PC9 cells than in the normal human bronchial epithelial cells. **f-h** qRT-PCR analysis of the levels of circCDR1, circASPH, and circNETO2 in the tissue samples of lung adenocarcinoma, respectively. Every test was carried out in triplicate and n = 3 for each cell group.

**Fig. S2 Correlation between circASPH expression level and clinical characteristics of lung adenocarcinoma. a** Representative FISH images showing circASPH expression in the adjacent normal tissues and cancerous tissues of lung adenocarcinoma. **b** CircASPH expression levels were higher in the patients with T2-3 stage than with T1 stage. **c** CircASPH expression levels were higher in the patients with the N1-2 stage than with N0 stage. **d** Kaplan-Meier analysis of the correlation between circASPH expression levels and the overall survival.

**Fig. S3 Direct regulation of circASPH by HMGA2 and the interaction between HMGA2 and Twist1.** **a-c** qRT-PCR, western blot, and ICC analysis of the expression levels of HMGA2 in the HMGA2-overexpressing and control PC9 cells. α-tubulin served as the internal control. **d** qRT-PCR analysis of the circASPH levels in HMGA2-overexpressing and control PC9 cells. **e** ChIP of HMGA2 in A549 cells, followed by RT-PCR to determine DNA enrichment in the promoter of ASPH gene. The schematic representation under the graph is the position of each amplicon relative to the TSS (n = 3 for each amplicon site). The amplified products of position -801 to -605 were further determined by agarose gel electrophoresis. **f** Coupling HMGA2 Co-IP with mass spectrometry to discover the HMGA2-interacting proteins. The full information are provided in Supplementary Table 2. **g** Western blot analysis of the HMGA2 and Twist1 protein in the Co-IP eluate. **h-j** Peptide sequence tags at m/z 918.43695, 1119.00391, and 1040.44312 of the STAT3, Twist1, and BTF3 protein, respectively. Every test was carried out in triplicate and n = 3 for each cell group.

**Fig. S4** **The direct regulation of circASPH by Twist1 is HMGA2-dependent.** **a-c** Verification of the overexpression of Twist1 in A549 cells. **d** qRT-PCR for the levels of circASPH in Twist1-overexpressing and control A549 cells. **e-g** Successful knockdown of HMGA2 mRNA and protein using a lentivirus vector which expresses specific siRNA to target HMGA2. **h** qRT-PCR analysis of the circASPH levels in the lenti-siHMGA2 and control A549 cells. **i-k** Verification of Twist1 overexpression in the lenti-siHMGA2 A549 cells using a Twsit1-expressing adenovirus vector. **l** qRT-PCR analysis of the circASPH levels in the HMGA2-knockdown A549 cells with or without Twist1 overexpressing. **m** ChIP of Twist1 in the normal and lenti-siHMGA2 A549 cells. The precipitated chromatin was PCR-amplified to amplify position -285 to -30. **n** qRT-PCR analysis of the levels of mature ASPH mRNA and ASPH pre-mRNA in the HMGA2-overexpressing and control A549 cells. **o** qRT-PCR analysis of the levels of mature ASPH mRNA and ASPH pre-mRNA in the Twist1-overexpressing and control A549 cells. Every test was carried out in triplicate and n = 3 for each cell group.

**Fig. S5 Decreased proliferation, migration, and invasion of A549 cells by circASPH silencing in vitro and verification of the overexpression of circASPH in A549 and PC9 cells.** **a** The schematic model of si-circASPH. **b** SiRNA specifically targeting the back-spliced junction site of circASPH successfully decreased the expression levels of circASPH in A549 cells. The expression levels of ASPH mRNA were not affected by the knockdown of circASPH. **c, d** DNA synthesis assessed using an EdU assay in the si-control and si-circASPH A549 cells. The quantified data are presented as the percentage of EdU-incorporated cells. Nuclei were stained with Hoechst 33342 (blue). **e, f** The migration of A549 cells was significantly decreased by circASPH knockdown. The migration distances (μm) of A549 cells were quantified. **g, h** The invasion of A549 cells was apparently decreased by circASPH knockdown. The quantified data are presented as the number of invading cells per HPF. HPF, high-power field. **i** Morphologies of si-circASPH A549 and PC9 cells were observed using a scanning electron microscopy assay. **j, k** Lenti-circASPH only enhanced the levels of circASPH in A549 and PC9 cells. The levels of ASPH mRNA were not affected by circASPH overexpression. Every cell test was carried out in triplicate and n = 3 for each cell group.

**Fig. S6** **MiR-370 inhibits the proliferation, migration, and invasion of A549 and PC9 cells in vitro.** **a** qRT-PCR analysis of the levels of miR-370 in the tissue samples of lung adenocarcinoma. **b** qRT-PCR analysis of the expression levels of miR-370 in A549, PC9, and BEAS-2B cells. **c** Verification of miR-370 overexpression in A549 and PC9 cells. Cells transfected with miR-Scramble served as the control cells. **d, e** EdU assay in the miR-370-overexpressing A549 and PC9 cells. The proliferation of miR-370-overexpressing A549 and PC9 cells was decreased compared with the control cells. **f-h** Assessment of migration of A549 and PC9 cells. The migration of miR-370-overexpressing A549 and PC9 cells was suppressed. **i, j** The invasion of miR-370-overexpressing A549 and PC9 cells was inhibited compared with the control cells. HPF, high-power field. Every test was carried out in triplicate and n = 3 for each cell group.

**Fig. S7** **The oncogenic effects of circASPH are HMGA2-dependent.** **a, b** The lenti-circASPH/siHMGA2 cells demonstrated reduced proliferation capacities. **c, d** The invasion of lenti-circASPH/siHMGA2 cells was decreased compared with the control cells. HPF, high-power field. **e-g** The knockdown of HMGA2 inhibited the migration of circASPH-overexpressing cells by wound scratch assay. **h-j** Xenograft tumor models show that tumors grown from the lenti-circASPH/siHMGA2 cells were smaller than those grown from the lenti-circASPH/siControl cells.
